# Observing the suppression of individual aversive memories from conscious awareness

**DOI:** 10.1093/cercor/bhae080

**Published:** 2024-06-11

**Authors:** Xuanyi Lin, Danni Chen, Jing Liu, Ziqing Yao, Hui Xie, Michael C Anderson, Xiaoqing Hu

**Affiliations:** Center for Sleep & Circadian Biology, Weinberg College of Arts and Sciences, Northwestern University, Evanston, IL, 60208, United States; Department of Neurobiology, Weinberg College of Arts and Sciences, Northwestern University, Evanston, IL, 60208, United States; Department of Psychology, The State Key Laboratory of Brain and Cognitive Sciences, The University of Hong Kong, Hong Kong SAR, China; Department of Psychology, The State Key Laboratory of Brain and Cognitive Sciences, The University of Hong Kong, Hong Kong SAR, China; Department of Applied Social Sciences, The Hong Kong Polytechnic University, Hong Kong SAR, China; Department of Psychology, The State Key Laboratory of Brain and Cognitive Sciences, The University of Hong Kong, Hong Kong SAR, China; Department of Psychology, The State Key Laboratory of Brain and Cognitive Sciences, The University of Hong Kong, Hong Kong SAR, China; MRC Cognition & Brain Sciences Unit, Behavioural and Clinical Neuroscience Institute, University of Cambridge, Cambridge, CB2 7EF, UK; Department of Psychology, The State Key Laboratory of Brain and Cognitive Sciences, The University of Hong Kong, Hong Kong SAR, China; HKU-Shenzhen Institute of Research and Innovation, Shenzhen, 518057, China

**Keywords:** EEG, multivariate pattern analysis, retrieval suppression, representation similarity analysis, Think/No-Think

## Abstract

When reminded of an unpleasant experience, people often try to exclude the unwanted memory from awareness, a process known as retrieval suppression. Here we used multivariate decoding (MVPA) and representational similarity analyses on EEG data to track how suppression unfolds in time and to reveal its impact on item-specific cortical patterns. We presented reminders to aversive scenes and asked people to either suppress or to retrieve the scene. During suppression, mid-frontal theta power within the first 500 ms distinguished suppression from passive viewing of the reminder, indicating that suppression rapidly recruited control. During retrieval, we could discern EEG cortical patterns relating to individual memories—initially, based on theta-driven visual perception of the reminders (0 to 500 ms) and later, based on alpha-driven reinstatement of the aversive scene (500 to 3000 ms). Critically, suppressing retrieval weakened (during 360 to 600 ms) and eventually abolished item-specific cortical patterns, a robust effect that persisted until the reminder disappeared (780 to 3000 ms). Representational similarity analyses provided converging evidence that retrieval suppression weakened the representation of target scenes during the 500 to 3000 ms reinstatement window. Together, rapid top-down control during retrieval suppression abolished cortical patterns of individual memories, and precipitated later forgetting. These findings reveal a precise chronometry on the voluntary suppression of individual memories.

## Introduction

Following an upsetting event, even seemingly innocuous reminders can bring us back to the traumatic scene in the blink of an eye, triggering intrusive memories and distress. When this happens, people often recruit inhibitory control to terminate retrieval and to keep unwelcome memories out of awareness, a process known as retrieval suppression (Anderson and Hulbert 2021; Engen and Anderson 2018). Although effective retrieval suppression is essential for cognitive functioning and mental well-being (Catarino et al. 2015; Hu et al. 2017; Mary et al. 2020), much remains unknown about its mechanisms. Indeed, no study has yet observed individual memories as they are intentionally suppressed, a prerequisite to tracking the dynamics of memory control.

Neuroimaging research suggests that during retrieval suppression, the prefrontal cortex exerts inhibitory control over the hippocampus and the medial temporal lobe to stop retrieval (Anderson et al. 2004; Depue et al. 2007; Mary et al. 2020). Top-down control further attenuates activity in neocortical areas implicated in memory reinstatement (Gagnepain et al. 2017). Given its limited temporal resolution, however, functional MRI precludes a detailed account of the temporal dynamics underlying the suppression of individual memories.

Conversely, research using EEGs can track the neurodynamics of retrieval suppression. Specifically, retrieval suppression enhanced the midfrontal N450 and theta power that are related to conflict monitoring and inhibitory control processes (Bergström et al. 2009; Mecklinger et al. 2009; Depue et al. 2013; Crespo-García et al. 2022). Moreover, suppressing unwanted memories attenuates EEG activity implicated in episodical retrieval and working memory maintenance, e.g. P300 and negative slow-waves (Hu et al. 2015; Hellerstedt et al. 2016). However, EEGs’ poor spatial resolution, together with a focus on univariate between-condition analyses, has rendered it difficult to isolate individual memories as they are suppressed. Recently, advances in multivariate analyses have allowed researchers to exploit EEG scalp distributions, aided by representational similarity analysis (RSA), to identify specific memory representations (Kriegeskorte et al. 2008; Wolff et al. 2017; Bae and Luck 2018). Here, leveraging EEGs’ temporal resolution and multivariate analyses, we sought to isolate cortical patterns unique to individual memories, and to observe how suppression abolishes those item-specific patterns.

For this purpose, we asked participants to retrieve or to suppress aversive scenes when confronting object cues in a Think/No-Think (TNT) paradigm (Küpper et al. 2014; Catarino et al. 2015). To track the suppression of individual memories, we took a 2-step approach in our multivariate EEG analyses. First, we used MVPA within each retrieval condition to isolate item-specific cortical EEG patterns and to examine their temporal development in relation to suppression. Next, we constructed subjective representational dissimilarity matrices (RDM) for both cues and aversive scenes, and then correlated these matrices with EEG-based decoding matrices using RSA. The RSA thus provides a powerful tool to investigate the representational nature of the item-specific decoding patterns (Grootswagers et al. 2019; Liu et al. 2023; Kriegeskorte et al. 2008; Proklova et al. 2019; Zhao et al. 2017).

We hypothesized that suppression will arise during 2 critical windows to stop retrieval: first, suppression will engage a rapid inhibitory control process well before the reminder elicits full-blown episodic recollection, i.e. to disrupt the cue-to-memory conversion process around 500 ms (Yaffe et al. 2014; Staresina and Wimber 2019; Treder et al. 2021). Second, given that hippocampus-driven pattern completion would reinstate neocortex-mediated memory traces during a 500 to 1500 ms window, suppression should attenuate and even abolish residual cortical reinstatements during 500 to 1500 ms. We further examined 4 to 8 Hz theta activity during the early 0 to 500 ms window between conditions, given the established role of frontal theta in cognitive control (Nigbur et al. 2011; Cavanagh and Frank 2014; Waldhauser et al. 2015; Crespo-García et al. 2022). By comparing how item-specific cortical patterns unfold over time between the retrieval and the suppression conditions, we gain a window into the timeline for how inhibitory control affects the recollection of individual memories.

## Materials and methods

### Participants

Recent emotional TNT (eTNT) research has typically recruited 18 to 25 participants, and yielded large effect sizes (*n* = 18 in Catarino et al. 2015; *n* = 24 in Gagnepain et al. 2017; *n* = 24 in Küpper et al. 2014; *n* = 18 in Liu et al. 2016; *n* = 25 in Zhang et al. 2016). A recent meta-analysis of TNT studies also revealed an effect size of 0.66 when using a direct retrieval suppression strategy among healthy participants (Stramaccia et al. 2021, page 839). We planned to recruit at least 36 participants, expecting a smaller effect size based on our pilot study using identical materials and procedures (*n* = 24). Sensitivity analysis revealed that a sample size of 36 would allow us to detect a moderate effect size of *dz* = 0.48 at 80% power, with a 5% false positive rate. With an expected exclusion rate of 10%, we recruited 41 participants (mean age = 19.57, age range: 18 to 23 yr, 26 females) from the University of Hong Kong. One participant was excluded because of noncompliance of task instructions, leaving 40 participants in the analyses. In the supplemental experiment, we additionally recruited 22 participants (mean age = 24.91, age range: 19 to 31 yr, 17 females) from the University of Hong Kong to obtain the subjective similarity ratings on the images used in the main experiment for RSA. Ethical approval was obtained from the Human Research Ethics Committee of The University of Hong Kong. All participants provided written consent prior to their participation.

### Materials and procedure

**Fig. 1:**
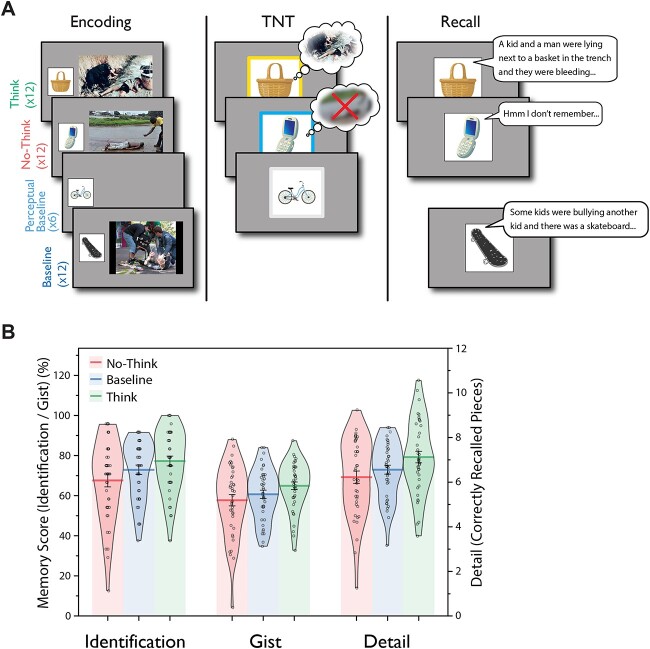
Experimental procedure and suppression-induced forgetting. A) The eTNT task included 3 phases. (i) Encoding: participants first learned object-aversive scene stimulus pairings; and they also viewed some objects without any paired scene (i.e. perceptual baseline). Scene images are from the International Affective Picture System (Lang et al. 2008); object images are from Brady et al. (2008); (ii) eTNT task: participants either retrieved (Think) or suppressed the retrieval (No-Think) of negative scene memories. Participants were also presented with Perceptual Baseline trials without any retrieval, i.e. no-retrieval; Think, No-Think, and Perceptual Baseline instructions were cued by a green, red, or blue colored box, respectively, surrounding the cue object; (iii) recall: participants viewed object cues and verbally described their associated scenes. During the TNT phase, we used yellow, blue, and white colored box surrounding the object cues to signal different conditions (Think, No-Think, Perceptual Baseline), with the colors being counterbalanced across participants. B) Suppression-induced forgetting on *Identification*, *Gist* and *Detail* measures from the recall test. Suppression-induced forgetting can be seen in the lower recall of No-Think than Baseline items (*n* = 40).

We used 42 object-scene picture pairs from Küpper et al. (2014). Scenes depict aversive contents such as natural disasters, assault, injury, etc (Lang et al. 2008). Each scene was rated by an independent sample of participants (*n* = 12) on a 9-point Likert scale for valence (1, extremely negative; 9 extremely positive) and arousal (1, extremely calm; 9 extremely exciting/arousing). Each object resembled an item from its paired negative scene, thus establishing naturalistic and strong associations. Six pairs were used for instruction and practice purposes. The remaining 36 pairs were equally divided into 3 sets, with 12 pairs in each of 3 following conditions: Think, No-Think, and Baseline. Pictures used in the 3 conditions were counterbalanced across participants and were matched on valence and arousal ratings (for valence, mean ± SD, 1.65 ± 0.06; 1.68 ± 0.07; 1.68 ± 0.07; for arousal: 5.11 ± 0.28; 5.10 ± 0.23; 5.07 ± 0.21). Another set of 6 objects without any paired scenes was used as Perceptual Baseline trials, which did not involve any memory retrieval. Participants completed the following sessions in order: Encoding, EEG-based TNT, and Cued Recall (Fig. 1A). At the end of the study, participants completed a 3-item, instruction compliance questionnaire. For transparent reporting, we also implemented an implicit affect task right after the TNT task to measure retrieval suppression’s aftereffect on spontaneous affective responses. These results are beyond the scope of the current study and will not be reported here.

### Encoding

Participants were presented with 42 object-scene pairings, plus 6 objects (without scene pairing) from the Perceptual Baseline condition. This ensured that participants would be familiar with all the objects during the subsequent TNT phase. Each object-scene pair was presented on an LCD monitor for 6 s with an inter-trial-interval (ITI) of 1 s. Participants were instructed to pay attention to all the details of each scene, and to associate the left-sided object and the right-sided scene. They next completed a test–feedback session, in which each object was presented for up to 4 s until participants pressed a button indicating whether they could recall the associated scene or not. If participants gave a “yes” response, they were presented with 3 scenes from the learning phase and needed to identify the correct one within another 4 s. Regardless of accuracy, the correct pairing was then presented for 2.5 s. This test–feedback cycle was repeated until participants reached 60% accuracy, and no limit on the number of cycles required to achieve this criterion was set. All participants reached 60% accuracy within 3 rounds: 26 participants reached this criterion within the first cycle, 13 participants within 2 cycles, and 1 in 3 cycles. Following the test–feedback cycles, participants completed a recognition-without-feedback test, so as to confirm that items from different conditions were encoded at comparable levels before the TNT phase (mean ± SD across 3 conditions are 86.0% ± 12.4%, *F*(73.4) = 73.40, *P* = 0.237; *M*_Think_ = 86.9%, *M*_No-Think_ = 84.2%, *M*_Baseline_ = 87.3%).

### Think/No-Think

Participants were presented with 24 objects from the 36 object-scene pairings, with 12 objects in each of the TNT conditions, respectively. The remaining 12 objects were not shown in the TNT and were used in the Baseline condition. These 24 objects were presented in either yellow- or blue-colored frames indicating Think and No-Think conditions, with colors being counterbalanced across participants. Six objects (without any pairing scenes) were presented in white-colored frames and served as Perceptual Baseline trials. Thus, 30 unique objects were shown in the TNT session. Each object was presented 10 times, resulting in 300 trials. Each trial began with a fixation cross (2 to 3 s), followed by the object in a colored frame for 3 s. The ITI was 1 s.

Across all 3 conditions, we instructed participants to fix their eyes on the object, and to never disengage attention from it during the whole 3-s presentation. For Think trials, participants were instructed to try their best to think about each object’s associated scene in detail, and to keep the scene in mind while the object remained on the monitor. For No-Think trials, participants were given direct-suppression instructions: they should pay full attention to the presented object while refraining from thinking about anything associated with it. If any thoughts or memories other than the object came to mind, they needed to try their best to push the intruding thoughts/memory out of their mind and refocus on the object. Participants were also prohibited from using any thought substitution strategies (i.e. thinking about a different scene). For Perceptual Baseline trials, participants were simply instructed to focus on the object throughout the whole trial.

### Cued recall

Following the TNT session, participants were presented with each of the 36 objects from *Think, No-Think*, and *Baseline* conditions. Each object was presented at the center of the monitor, alongside a beep sound prompting participants to verbally describe the associated scenes within 15 s. The ITI was 3 s. Participants’ verbal descriptions were recorded for later scoring. Perceptual Baseline objects were not shown in this recall test because they were not paired up with any scenes.

### Subjective dissimilarity rating

To obtain measures of subjective dissimilarity ratings of cue objects and target scenes for RSA analysis, an independent sample of participants gave pairwise (dis)similarity ratings for the cue (object) and target (scene) images regarding their *perceptual* and *conceptual* dissimilarities. Participants gave ratings in a 12 by 12 matrix for the 12 images in each of the Think and No-Think conditions, and in a 6 by 6 matrix for the 6 images in the Perceptual Baseline condition. Images were presented on the top row and the most left column, with columns/rows representing the same 12 (or 6) images. Participants rated the perceptual and conceptual dissimilarities between every possible stimulus pairing, within the cue category and the target category, separately. Participants rated along the scale from 1 (extremely dissimilar) to 9 (extremely similar).

### Cued recall analyses

Two trained raters who were blind to experimental conditions coded each of the verbal descriptions along 3 dimensions following the criteria used in a previous study (Küpper et al. 2014), namely, *Identification*, *Gist*, and *Detail*. Each measure focused on different aspects of memories: Identification referred to whether the verbal description was clear enough to correctly identify the unique scene, and was scored as 1 or 0. Inconsistent ratings were resolved by averaging 0 and 1, resulting in a score of 0.5. Gist measured whether participants’ verbal descriptions contained critical elements pertaining to the scene’s main themes. Two independent raters identified 2 to 4 gists for each scene (Küpper et al. 2014). We scored *gist* as proportion, using the number of correct gists from participants’ verbal reports divided by all possible gists for each scene. The Detail score measured how many correct meaningful segments were provided during the verbal description, and were scored on the number of details. Interrater agreement for the scoring of all 3 measures was above 0.7, which was considered as acceptable (Blick et al. 2018): Identification *r* = 0.71, Gist *r* = 0.90, Detail *r* = 0.86.

We conducted separate 1-way analyses of variance (ANOVAs) with 3 within-subject conditions (Think vs. No-Think vs. Baseline) on the percentage of Identification, Gist, and Details. We then examined the suppression-induced forgetting effect by conducting planned pairwise *t*-tests between No-Think and Baseline, with a negative difference (i.e. No-Think minus Baseline) indicating below-baseline, suppression-induced forgetting.

We report findings with *P* < 0.05 as significant. Within-subject ANOVAs are reported with Greenhouse–Geisser corrected *P*-values whenever the assumption of sphericity was violated. We report Cohen’s *dz* as effect size given our within-subject design (Lakens 2013).

### E‌EG recording and preprocessing

Continuous EEGs were recorded during the TNT session using ANT Neuro eego with a 500 Hz sampling rate (ANT, The Netherlands), from 64-channel ANT Neuro Waveguard caps with electrodes positioned according to the 10 to 5 system. The AFz served as the ground and CPz was used as the online reference. Electrode impedances were kept below 20 kilo-ohms before recording. Eye movements were monitored through EOG channels.

Raw EEG data were preprocessed in MATLAB using EEGlab Toolbox (Delorme and Makeig 2004) and ERPlab Toolbox (Lopez-Calderon and Luck 2014): data were first downsampled to 250 Hz, and were band-pass filtered from 0.1 to 60 Hz, followed by a notch filter of 50 Hz to remove line noise. Bad channels were identified via visual inspection, and were removed and interpolated before re-referencing to common averages. Continuous EEG data were segmented into −1000 to 3500 ms epochs relative to the cue onset, and baseline corrected using −500 to 0 ms as baseline period. Next, independent component analyses were implemented to remove eye blinks and muscle artifacts. Epochs with remaining artifacts (exceeding ±100 μV) were rejected. The numbers of accepted epochs used in all following analyses were comparable across Think (Mean ± SD, 100.33 ± 11.57) and No-Think (103.18 ± 10.61) conditions. Valid trials number in Perceptual Baseline is 56.58 ± 3.23. All EEG analyses were based on 61 electrodes, excluding EOG, M1, M2, AFz (ground) and CPz (online reference).

### Univariate event-related potentials and time frequency analyses

Six electrode clusters were selected for event-related potentials (ERP) and for Time Frequency analyses: left parietal (CP3/5, P3/5), parietal (Pz, CP1/2, P1/2), right parietal (CP2/4, P2/4), frontocentral (FC1/2, C1/2, FCz, Cz), left prefrontal (AF3, F3/5), and right prefrontal (AF4, F4/6).

We focused on a priori defined ERP components based on previous ERP-TNT research (e.g. Bergström et al. 2009; Chen et al. 2012; Depue et al. 2013; Hellerstedt et al. 2016; Hu et al. 2015; Mecklinger et al. 2009). These ERP components and time windows are: the frontocentral N450 (300 to 500 ms), taken to be sensitive of inhibitory control, and the parietal P300 (300 to 800 ms), indicative of episodic recollection. We calculated average amplitude from the time window, and submitted the amplitudes to paired *t*-tests between No-Think and Perceptual Baseline trials.

Time frequency transformation was performed in Fieldtrip toolbox (Oostenveld et al. 2011). Frequencies of interest increased logarithmically from 2.8 to 30 Hz, resulting in 22 frequency bins. Wavelet cycles increased linearly along with frequencies from 3 to 7, with decibel baseline normalization using power on −500 to −200 ms. We focus on the early theta power change on 200 to 400 ms which is indicator of inhibitory control (Nigbur et al. 2011; Cavanagh and Frank 2014), and theta and alpha power change on a post hoc late time window (500 to 3000 ms) following condition level decoding results.

Early theta power at each electrode was compared between No-Think and Perceptual Baseline after averaging on 200 to 400 ms across 4 to 8 Hz, and then cluster corrected according to electrode positions in Fieldtrip (Oostenveld et al. 2011). The suppression-associated reduction of theta and alpha power on later time window was examined by averaging on 500 to 3000 ms across 4 to 8 Hz (theta) and 9 to 12 Hz (alpha), and then compared between No-Think and Think/Perceptual Baseline with neighbor cluster correction in Fieldtrip. The channel neighbors were defined in the same way as in channel searchlight analysis.

### Condition-/item-level decoding with time domain EEG

Decoding analyses were conducted in MATLAB using scripts adapted from Bae and Luck (2018), which used a support vector machine (SVM) and error-correcting output codes (ECOC). The ECOC model combined results from several binary classifiers for prediction output in multiclass classification. The preprocessed EEGs were PCA-decomposed (Fieldtrip toolbox, Oostenveld et al. 2011), with the first 15 PCA components as features in the decoding analyses. PCA decomposition was applied to reduce potential overfitting in classifier training because of the large number of features, i.e. number of channels (Jollans et al. 2019).

In the condition-level decoding, we used 1-vs.-1 SVMs to perform pairwise decoding among the 3 conditions (Think vs. Perceptual Baseline, No-Think vs. Perceptual Baseline, and Think vs. No-Think). For Think vs. Perceptual Baseline and No-Think vs. Perceptual Baseline condition-level decoding, we first subsampled trials in T/NT to be comparable with Perceptual Baseline so that each condition had about 56 trials. Next, EEG trials from each condition were randomly divided into 10 equal sets and were averaged within each set into sub-ERPs to improve signal-to-noise ratio. The decoding was achieved within each participant from −500 to 3000 ms using these sub-ERPs in a 10-fold cross validation: each time 9 of the 10 sub-ERPs are used as training data set with the condition labels, and the remaining one was used as testing data set. After splitting training and testing data sets, sub-ERPs were both normalized using the mean and standard deviation of training data set to remove ERP-related activity. This process was conducted on every 20 ms time point with the surrounding ±10 ms data averaged, and repeated for 10 iterations to minimize the impact of random train–test splitting. We were comparing condition-level decoding accuracy against its chance level, 50%, given our pairwise, binary decoding. The use of 1/number of classes as the chance level might not always be appropriate (Combrisson and Jerbi 2015). Here, we used the pre-stimulus baseline period of each epoch in a classification analysis to estimate the chance level, given that the baseline data should not contain any condition-related information. The estimation showed that the chance levels in each pair-wise condition-level decoding were all around 50%: 49.98% ± 0.66% in retrieval vs. no-retrieval; 50.06% ± 0.61% in suppress vs. no-retrieval, and 49.99% ± 0.70% in retrieval vs. suppress. We thus continued to use 50% as the chance level in our condition-level decoding analysis.

For item-level decoding, we used 1-vs.-all SVMs to decode individual items within each condition, separately. Decoding procedures were the same as condition-level decoding. Thus, the trial numbers of each stimulus are first matched to the least one within each participant (at most 10 trials for each stimulus, if no trial was rejected in preprocessing). Then, the total trials of each stimulus were randomly divided into 3 sets before averaging and before the 3-fold cross validation. Both training data set and testing data set were normalized using the mean and standard deviation of training data set. The decoding process was conducted on every 20 ms time point with the surrounding ±10 ms data averaged and was repeated for 10 iterations to minimize the impact of randomization (results remained similar when using 100 iterations, see Fig. S2a). The chance levels of item-level decoding were estimated using the baseline periods as described in the condition-level decoding. We used 1/number of items as the chance level in statistical comparisons since the estimations were highly similar to 1/number of items: 8.32% ± 0.27% in Think, 8.34% ± 0.28% in No-Think, and 16.54% ± 0.58% in Perceptual Baseline. For Think and No-Think conditions, the chance levels were 1/12 (8.33%). For Perceptual Baseline trials, the chance level was 1/6 (16.67%).

Given different numbers of items in Perceptual Baseline (6 items) and TNT conditions (12 items), we conducted a resampled decoding in Think and No-Think, respectively. During each iteration we randomly selected 6 out of all 12 items from TNT conditions, before dividing and averaging into 3 sets. Considering the randomization used only half of the items, we increased iterations to 20 times. An item-level decoding with 20-iterations was also repeated in Perceptual Baseline. This allows direct comparisons between the item-level decoding accuracy in TNT with Perceptual Baseline.

Following the statistical analysis procedure in Bae and Luck (2018), decoding accuracy at each time point within the 0 to 3000 ms time window was compared with chance level by 1-tailed paired *t*-tests. Multiple comparisons were controlled by nonparametric cluster-based Monte Carlo procedure. Specifically, a null distribution was constructed by assigning trial level classification results to random classes (as if the classifier has no knowledge of actual information), and then timepoint-by-timepoint *t*-tests were performed to obtain a maximum summed *t*-value of continuous significant time cluster, which then repeated for 1000 times. The resulting null distribution contained 1000 summed *t*-values, which would be the distribution of the cluster summed-*t*s when there is no true difference between decoding results and chance level. Both the cluster alpha and the alpha to obtain critical values from the permutation null distribution were set at 0.05 (on the positive tail, 1-tail against chance).

The condition-level decoding accuracy along time were similar, except that the null distribution was constructed by randomly assigning condition labels to trial level classification results with 2-tail repeated measure *t*-test and clusters were obtained on positive/negative tails, respectively. Thus, the critical values from the permutation null distribution were at 2.5% on the negative clusters null distribution and 97.5% on the positive clusters null distribution.

### Condition-/item-level decoding with time-frequency domain EEG

Time domain PCA-decomposed EEG was wavelet transformed into time-frequency domain data in Fieldtrip Toolbox (Oostenveld et al. 2011) before decoding, using the same parameters as described in the time frequency analyses. Then the decoding was conducted for each frequency bin data across time in the same procedure as in time-domain decoding (treating each frequency bin data as time domain data).

The statistical analyses for time-frequency domain decoding were similar to those of time domain decoding, except that here clusters were calculated in a 2D matrix instead of on a 1D time axis, and the cluster alpha was set at 0.05. Also, observed clusters were compared with the null distribution clusters of the same rankings. The statistical comparison of a single time-frequency decoding was performed against chance level (1-tailed), and that of the difference between 2 time-frequency decoding was performed against 0 (2-tailed). Theta (4 to 8 Hz) and alpha (9 to 12 Hz) decoding were assessed after averaging across the corresponding frequency bin.

### Channel searchlight decoding

Since both condition- and item-level decoding used PCA-decomposed EEGs, to examine which electrodes contributed the most to the decoding accuracy, we conducted a channel searchlight decoding using subsets of the 61 channels as features (Treder 2020).

Specifically, we first divided all channels into 61 neighborhoods, centering each channel according to its location (conducted in Fieldtrip Toolbox, Oostenveld et al. 2011, via ft_prepare_neighbors() function using “triangulation” method). Immediately neighboring channels were clustered together, resulting in 6.39 ± 1.50 channel neighbors for each channel (with overlaps). Then the time domain EEG was averaged on time windows of interest, i.e. averaged on 0 to 500 ms, 500 to 3000 ms, etc., to inspect the decoding topographical distribution on different time windows. The rest of the procedure was the same as time domain EEG decoding: we divided data into 3 sets and averaged within each set before splitting training and testing data sets; then we normalized them using mean and standard deviation of training sets. Finally, the decoding was conducted with a 3-fold cross validation and 10 iterations. Theta/alpha searchlight was conducted in the same way as time-domain searchlight, after averaging time-frequency power on respective oscillation range (theta: 4 to 8 Hz; alpha: 9 to 12 Hz).

We compared channel searchlight topographies between item-level decoding in Think and No-Think with a 2-tailed paired-sample *t*-test at each channel. The multiple comparisons were controlled by cluster correction of channel neighbor clusters in Fieldtrip (Oostenveld et al. 2011). The neighborhood was defined in the exact same way as the channel searchlight analysis. Cluster alpha was set at 0.05. Observed clusters were compared with null distribution on positive/negative tails, respectively.

### High- vs. low-suppression grouping

We divided 40 participants into High- vs. Low-Suppression Groups, with 20 participants in each group based on the median split of No-Think-minus-Baseline Detail scores ranking. We used Detail because it captured both variability and suppression effects to a greater extent than did Identification (limited variability since it was a dichotomous measure) and Gist (did not show the suppression effect). Importantly, pre-TNT learning (i.e. recognition accuracies) was not different between the 2 subgroups (*t*(38) = 1.66, *P* = 0.105). Moreover, a 3 (within-participants: TNT conditions) by 2 (between-participants: subgroup) ANOVA on pre-TNT recognition accuracies showed no significant main effects or interactions (*P*s > 0.104). Thus, pre-TNT memory performance was comparable across the 2 subgroups and across TNT conditions.

Decoding accuracy at each time point during 0 to 3000 ms was compared between High- and Low-Suppression groups using 2-tail independent *t*-test. The null distribution was constructed by randomly assigning group labels to each subject before by-timepoint *t*-test, to obtain the max summed-*t* of continuous significant time cluster when group labels are randomized, which repeated for 1,000 times. The resulting 1,000 summed-*t* values would be the null distribution when no true difference exists between the 2 groups. Critical values from the permutation null distribution were at 2.5% on the negative clusters null distribution and 97.5% on the positive clusters null distribution (2-tail, *α*s = 0.05).

### Correlation analyses

We calculated Spearman’s Rho for all brain-behavior correlations. For memory, we reported the correlation with *Details* scores in main text, given it is a continuous memory measure and is more sensitive in capturing individual differences. The same correlation analyses with *Identification* and *Gist* measures were provided in supplementary. In condition-level decoding, memory of Think and No-Think was normalized by subtracting and then divided by Baseline memory. For condition-level time domain decoding, we focused on 500 to 3000 ms given our a priori hypothesis that neural activity between this time window would reflect retrieval of individual memories. To investigate the time course of these correlations, we also calculated Spearman’s Rho at each time point. In item-level time-domain decoding, we investigated the correlations between decoding accuracy and absolute memory score of the same condition during 500 to 3000 ms.

The cluster correction for correlation time course was performed: we first transformed Spearman’s Rho back to *t*-values to obtain the observed time-course clustered *t*-values and a null distribution. The null distribution was obtained by randomizing labels of the 2 variables of interest before calculating the Spearman’s Rho and corresponding *t*-value. The cluster alpha was set as 0.05, and the observed clusters were calculated for positive and negative clusters, respectively. The critical values of null distribution were at the 2.5% on both tails. The comparison between 2 correlation coefficients was conducted through a 2-sided *z*-test controlling for dependence (Lenhard and Lenhard 2014).

### Representation similarity analysis

For cue (object) and target (aversive scene) pictures, we averaged the subjective dissimilarity ratings from all 22 participants. This resulted in a perceptual and a conceptual RDM for cues and target pictures, respectively (Zhao et al. 2017; Zabicki et al. 2019). To obtain the neural RDM, we performed a pairwise item-level decoding using PCA-decomposed EEGs (Grootswagers et al. 2019; Proklova et al. 2019). The decoding procedure was the same as described in the time-domain decoding, except that the classifier training and classification was performed between every 2 stimuli. This resulted in neural RDMs at every timepoint. We then correlated this neural RDM with the cue- and target-RDMs derived from subjective dissimilarity rating, and derived a time course of the correlation coefficients using Spearman’s Rho. Note that because this RSA tests the *a priori* hypotheses regarding the cue-target retrieval processes, the significance of the Spearman’s Rho was obtained via 1-sided test, and was tested during time window showing significant item-level decoding without corrections. We repeated this analysis for each of the Think, No-Think, and Perceptual Baseline conditions, and on perceptual and conceptual ratings, respectively.

## Results

### Suppressing retrieval induces forgetting of emotional memories

Following the eTNT task, participants completed a cued recall test during which they verbally described the aversive scene that they thought was linked to each of the cue objects. We coded and scored verbal descriptions on *Identification, Gist*, and *Detail* (see Materials and Methods). Each of these 3 scores was submitted to a 1-way repeated-measure (Think, No-Think, and Baseline) ANOVA. Results showed a significant condition effect on Identification (*F*(1.87,72.93) = 7.35, *P* = 0.002, *η*_p_^2^ = 0.159); Detail (*F*(1.93,75.2) = 13.79, *P* < 0.001, *η*_p_^2^ = 0.261); and Gist (*F*(1.92,74.95) = 6.22, *P* = 0.004, *η*_p_^2^ = 0.138). Planned contrasts (Baseline vs. No-Think) showed significant suppression-induced forgetting on Identification, *t*(39) = −2.07, *P* = 0.045, *dz* = 0.33, and on Details, *t*(39) = −2.16, *P* = 0.037, *dz* = 0.34, whereas the forgetting effect on Gist did not reach significance, *t*(39) = −1.58, *P* = 0.123, *dz* = 0.25, see Fig. 1B.

### Stopping retrieval is distinct from not-retrieving: univariate and multivariate condition-level EEG analyses

We first analyzed well-established ERPs reported in prior research on retrieval suppression (e.g. N450, P300). Consistent with previous findings, retrieval suppression (vs. no-retrieval, i.e. the Perceptual Baseline condition) significantly enhanced frontocentral N450 (*t*(39) = 3.28, *P* = 0.002, *dz* = 0.52) and reduced central-parietal P300 activities (*t*(39) = 4.33, *P* < 0.001, *dz* = 0.69, see Fig. S1). Visual inspection of the ERPs suggests that the TNT conditions modulated the negative slow waves over prefrontal electrodes. Indeed, paired-sample *t*-tests showed that No-Think trials significantly reduced the NSW than Perceptual Baseline trials over the left prefrontal electrodes, *t*(39) = −2.69, *P* = 0.010, *dz* = 0.43 (Fig. S1). Our ERP results (e.g. N450, P300, NSW) are highly consistent with existing TNT literature (e.g. Depue et al. 2013; Hu et al. 2015; Waldhauser et al. 2015; Hellerstedt et al. 2016; Satish et al. 2022). Thus, we mainly focus on the results from the multivariate decoding analyses.

**Fig. 2:**
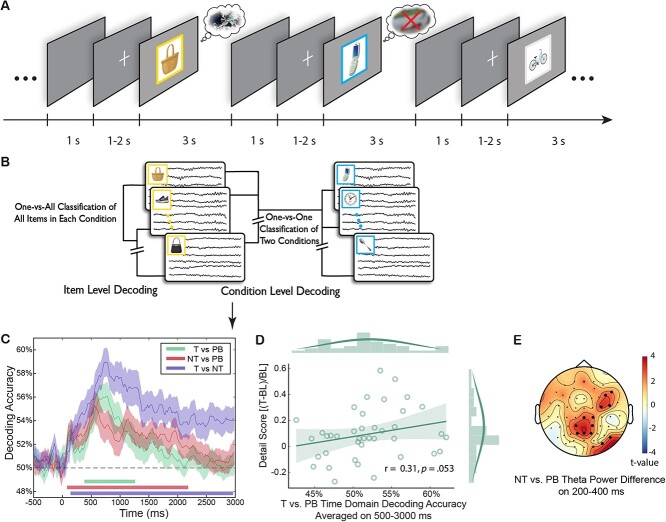
Decoding approaches diagram and condition-level time-domain EEG decoding results. A, B) An illustration of trial flow in the EEG-based eTNT task, and the logic of decoding analyses. C) Condition-level decoding based on time domain EEGs revealed significant differences in all 3 pairwise comparisons. Colored lines along *x* axis indicate significant clusters (permutation cluster corrected): No-Think vs. Perceptual Baseline, 80 to 2180 ms, *P*_corrected_ < 0.001; Think vs. Perceptual Baseline, 380 to 1640 ms, *P*_corrected_ < 0.001; Think vs. No-Think, 140 to 2960 ms, *P*_corrected_ < 0.001. Shaded areas indicate standard errors of the mean (S.E.M). D) Time domain Think vs. Perceptual Baseline decoding accuracies during the 500 to 3000 ms window was positively correlated with the above-baseline enhancement of memory recall in the Think condition, based on the *Detail* score, (Think − Baseline)/Baseline, in the final cued recall test, i.e. the retrieval benefit, proportional to baseline. E) Retrieval suppression elicited stronger theta power than no-retrieval during 200 to 400 ms (NT > PB). The decreased theta power showed a frontal-central distribution. Significant electrodes were cluster corrected and are highlighted.

The between-condition ERP differences are corroborated by between-condition multivariate analysis. The condition-level multivariate decoding not only distinguished retrieval suppression from voluntary retrieval (NT vs. T, *P*_corrected_ < 0.001, Fig. 2C, purple), but also from no-retrieval (NT vs. PB, *P*_corrected_ < 0.001, Fig. 2C, red). These differences imply that distinct cognitive operations contributed during retrieval suppression compared with either voluntary retrieval or no-retrieval. Differences between No-Think and Think conditions emerged as early as 140 ms and persisted throughout the entire trial period until ~3000 ms. In addition, we also could distinguish retrieval from no-retrieval (T vs. PB, *P*_corrected_ < 0.001, Fig. 2C, green). We also found that the latter 500 to 3000 ms T vs. PB decoding accuracies tended to predict better scene retention of the Think items in later *Detail recall, r* = 0.31, *P* = 0.053 (Fig. 2D, though the result was not statistically significant), and they significantly predicted *Identification*, *r* = 0.36, *P* = 0.022 (Fig. S2b). These results suggested that at least some of the latter T vs. PB decoding differences were associated with active retrieval processes during the Think condition.

Retrieval suppression could also be distinguished from retrieval and passive viewing based on time-frequency domain EEGs. The between-condition decoding results revealed differences among all pairwise comparisons (Fig. S2c and d). Consistent with an early, active control process associated with suppression, we found, within the first 500 ms, significant NT vs. PB decoding in 4 to 8 Hz theta activity over the frontal and posterior regions, which continued throughout the 3000 ms epoch (Fig. S2e). Time-frequency results showed that, during the 200 to 400 ms window, retrieval suppression (vs. retrieval or no-retrieval) led to enhanced midline and right prefrontal theta power (NT > PB, *P*_corrected_ = 0.002, Fig. 2E; NT > T, *P*_corrected_ = 0.007, Fig. S4g). After this early theta enhancement, suppression was associated with reduced mid-frontal theta power from 500 to 3000 ms (NT < PB, theta: *P*_corrected_ < 0.001, Fig. S3b; NT < T, theta: *P*_corrected_ = 0.004, Fig. S4b, see Fig. S6 for the time-frequency representations in each condition).

Retrieval suppression also could be distinguished based on alpha activity, and such effects were enduring. Indeed, 9 to 12 Hz alpha activity drove condition-level decoding performance between 500 and 3000 ms (Fig. S2d) with retrieval suppression reducing alpha (NT < PB, *P*_corrected_ = 0.002, NT < T, *P*_corrected_ < 0.001, Figs. S3 and S4). Together, these findings suggest that increases in early theta power and reductions in later theta/alpha power may be hallmarks of active suppression that make it qualitatively distinct from simply not-retrieving.

### Spatial patterns in EEG discern individual episodic memories during retrieval: multivariate item-level EEG analyses

**Fig. 3:**
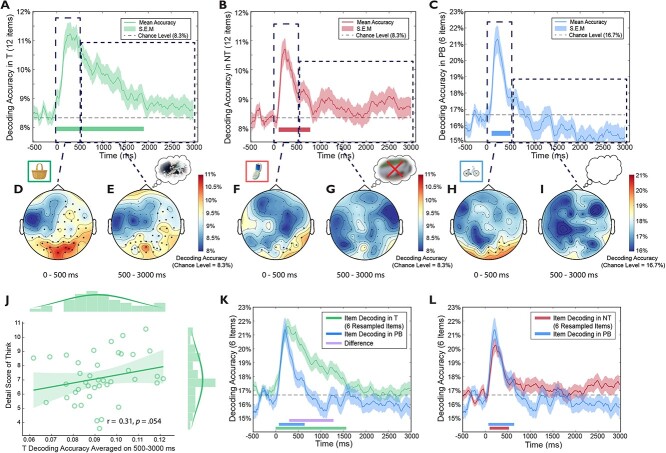
Item-level time domain decoding. A to C) The item-level decoding patterns (averaged across participants) in each retrieval condition. Lines at the bottom indicate significant time clusters against chance level, with permutation cluster correction (*α*s = 0.05). D to I) Channel searchlight analyses of time domain decoding during an early (0 to 500 ms) and a later time window (500 to 3000 ms). The color bar indicates decoding accuracy. Electrodes with significant decoding accuracies are highlighted (permutation cluster corrected, *α*s = 0.05). J) During Think trials, decoding accuracies averaged on 500 to 3000 ms predicted the number of details recalled from emotional scenes. K) Item-level decoding in the Think condition (using 6 resampled items) is higher than it is in the Perceptual Baseline condition from 300 to 1280 ms, *P*_corrected_ < 0.001. Lines at the bottom indicate cluster-corrected significant time clusters against the chance level (green and blue for think and perceptual baseline) or the difference between the 2 conditions (purple). l) Item-level decoding in the No-Think condition (using 6 resampled items) is not significantly different from decoding in the Perceptual Baseline condition. Lines at the bottom indicate significant time clusters against the chance level (red and blue for No-Think and Perceptual Baseline conditions, respectively).

Observing the suppression of individual memories requires an index sensitive to brain activity unique to each memory item so that the impact on suppression on that index may be tracked. We hypothesized that the spatial-temporal pattern of scalp-EEG as participants thought about each scene may contain information sufficient to distinguish that specific scene from all the others. To test this hypothesis, we performed a decoding analysis on PCA-decomposed EEG during Think trials, during which participants actively reinstated associated scenes. Consistent with our hypothesis, time-domain EEGs distinguished between individual scene memories across 0 to 1900 ms (Fig. 3A, *P*_corrected_ < 0.001). In sharp contrast, for Perceptual Baseline trials, above-chance decoding of individual items arose only in the 0 to 500 ms (to be precise, 80 to 480 ms, *P*_corrected_ = 0.010), but not in the 500 to 3000 ms window (Fig. 3C). To directly compare item-level decoding between retrieval and PB, we repeated the analyses with 6 randomly sampled items from the Think condition, to match the item number in the Perceptual Baseline (see Materials and Methods). We found that Think trials showed higher item-level decoding accuracies than Perceptual Baseline trials during the 300 to 1280 ms window (*P*_corrected_ < 0.001, Fig. 3K, purple lines).

Successful decoding of individual items in the early time window (e.g. 0 to 300 ms) likely reflects visual processing of unique object retrieval cues, which are present both for the object-scene pairs used in the Think condition, and in the single objects used in the PB condition. In the subsequent 300 to 1280 ms time window, however, higher decoding during Think trials would need to be driven by an item-specific processing present in the Think condition but not in the no-retrieval, PB condition. One possibility is that this later item-specific effect in the Think condition may reflect the reinstatement and maintenance of unique unpleasant scenes associated to the object cue, which may have gradually begun to emerge in awareness as they were recollected. Another possibility, however, is that item-level decoding in the Think condition may simply reflect more sustained attention to unique object cues in that condition, relative to the PB condition, for which participants may have correctly concluded that retrieval was unnecessary.

To distinguish these possibilities, we examined scalp regions giving rise to above-chance decoding during Think trials using searchlight decoding (see Materials and Methods). If greater decoding of individual items in the Think condition reflected sustained visual processing of object cues, successful decoding may be restricted to regions involved in object perception. Indeed, during the first 500 ms, occipital EEGs primarily drove the significant decoding in general, consistent with a primary role of visual-perceptual cue processing (Fig. 3D). In contrast, during the latter 500 to 3000 ms interval, significant decoding rested on a distributed set of regions implicated in memory retrieval such as the right prefrontal and parietal-occipital cortex (Fig. 3E). This finding suggests that item-level decoding beyond the first 500 ms is not dominated by object cue attention, but rather by the reinstatement of the associated scene memories. Converging with this possibility, item-level decoding accuracies during the latter 500 to 3000 ms time window tended to predict better scene memory on the *Detail* measure (though the correlation was not significant, *r* = 0.31, *P* = 0.054, Fig. 3J), whereas decoding during the early 0 to 500 ms time window did not (*r* = −0.10, *P* = 0.547).

Unlike during Think trials, the same searchlight analysis during Perceptual Baseline trials showed that significant decoding in the 0 to 500 ms window arose over a small cluster of occipital electrodes. The restriction of decoding success to occipital cortex suggests that classification hinged on visual object processing during that period (Fig. 3H). After this initial window, the latter part of the trial from 500 to 3000 ms showed no significant decoding at any electrode (Fig. 3I).

In sum, during retrieval, time-resolved EEG patterns suggest a staged cued-recall process: during the 0 to 500 ms window, EEG patterns could discern perceived items over occipital regions; during 500 to 3000 ms, EEG patterns could distinguish among retrieved items over frontoparietal-occipital regions. Furthermore, higher item-level decoding accuracies tended to predict better scene memory only in this latter, 500 to 3000 ms time window.

### Suppressing retrieval weakens and abolishes item-specific cortical patterns: evidence from multivariate EEG item-level analyses

Having established that the retrieval of individual scene memories can be indexed and tracked, we next sought to use this index to determine how and when suppression affected cortical patterns relating to individual memories. To this end, we examined whether retrieval suppression modulated item-specific cortical EEG patterns, particularly during the time window implicated in episodic retrieval.

We hypothesized that item-level decoding during No-Think trials would be possible initially, as participants focused their attention on the visually unique reminder cues, but that suppression would limit successful decoding throughout the remainder of the trial. Indeed, in the No-Think condition, item-level decoding accuracy was above chance from 100 ms, and remained so until 780 ms (*P*s_corrected_ = 0.002, Fig. 3B); decoding accuracy then dropped to chance-levels for the remainder of the 3000 ms. Consistent with the Think and Perceptual Baseline analyses, we used a priori defined time windows from 0 to 500 and 500 to 3000 ms to characterize the EEG scalp distributions contributing to decoding success. During the 0 to 500 ms window, item-level decoding was driven by occipital activity, resembling the EEG distributions found in the Perceptual Baseline condition during the same window (Fig. 3F and H). Strikingly, during the 500 to 3000 ms, there were no brain regions that contributed significantly to item-level decoding (Fig. 3G), suggesting that suppression had abolished evidence for cortical reinstatement of scene memories.

In addition to scalp EEG distributions revealed by the channel searchlight analysis, confusion matrices of item-level decoding provided converging evidence supporting the hypothesized stages of retrieval suppression: we observed significant above-chance item-specific classifications in all 3 conditions during the first 500 ms, when cue-processing might be expected to predominate; in contrast, distinctive classification patterns remained only in the Think condition during 500 to 3000 ms (Fig. S7b). Thus, suppression reduced cortical patterns during No-Think trials to the extent that they were as uninformative as items in our perceptual baseline condition, in which no scene retrieval was possible.

**Fig. 4:**
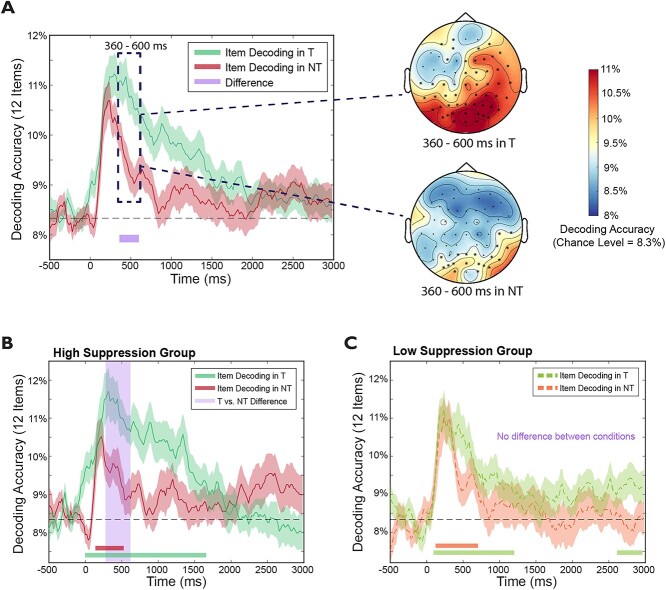
Item-level decoding results in *high-* and *low-suppression groups.* A) Retrieval suppression significantly reduced item-level decoding accuracies from 360 to 600 ms compared with retrieval (NT < T), with the right panel showing channel searchlight analyses on this time window. B, C) Comparisons between Think and No-Think item-level decoding in high-/low-suppression groups, respectively. In the *high-suppression group*, the Think vs. No-Think difference was significant during 280 to 620 ms, whereas no differences were found in the *low-suppression group*. Purple shades denote significant time clusters between conditions/groups (permutation corrected, 2-sided *α*s = 0.05).

To precisely characterize the temporal dynamics of retrieval suppression, we contrasted the time-dependent evolution of item-specific cortical patterns between retrieval suppression and both the retrieval and no-retrieval/perceptual baseline conditions. A direct comparison of Think vs. No-Think item-level decoding revealed that retrieval suppression reduced decoding accuracies from 360 to 600 ms (*P*_corrected_ = 0.027, Fig. 4A, left panel). Searchlight analyses during 360 to 600 ms revealed that, whereas voluntary retrieval engaged item-specific brain activity over frontal-parietal-occipital regions, retrieval suppression was only associated with occipital activity (Fig. 4A, right panel). When No-Think trials were directly compared with Perceptual Baseline trials (using 6 randomly sampled items from the No-Think condition), none of the differences survived permutation-based significant tests during the entire 0 to 3000 ms (see Fig. 3L).

Reduced decoding accuracy for individual No-Think items in the 360 to 600 ms window suggests that the retrieval stopping process may begin to exert its first effects within this window, a possibility consistent with findings from our condition-level decoding analyses. We next sought to determine whether prefrontal-control processes were linked to suppressed item-level decoding. Indeed, we found that in the No-Think (vs. Think) trials, this reduced item-level decoding was preceded by enhanced 200 to 400 ms theta power over midline and right prefrontal cortex (Fig. 2E).

Together with the evidence for suppression-specific patterns in the condition level analysis, these item-level decoding results reveal a precise timeline of how retrieval suppression unfolded: inhibitory control was engaged within the first 500 ms upon encountering an unwelcome reminder cue, presumably before the cue-to-memory conversion process completed, to obstruct retrieval and prevent reinstatement from happening. This early control weakened, and eventually abolished memory-specific cortical patterns during 500 to 3000 ms.

### Rapid suppression of individual memories led to their forgetting

To understand how the temporal dynamics of retrieval suppression influenced later forgetting of suppressed memories, we divided participants into High- vs. Low-Suppression groups based on a median-split of suppression-induced forgetting scores. We focused on below-baseline forgetting using our detail measure of scene recall (i.e. BL-minus-NT *Detail* scores, with a higher score indicating higher forgetting). Note that when using scores controlling for overall memory performance, i.e. (BL-minus-NT)/BL, participants assigned into High- vs. Low-Suppression groups were the same. We tested the hypothesis that successful suppression-induced forgetting was associated with a greater reduction in decoding accuracy (i.e. NT < T), compared with unsuccessful forgetting. In the High-Suppression group, suppression significantly reduced item-specific decoding accuracy in No-Think (vs. Think) trials during 280 to 620 ms (*P*_corrected_ = 0.017, Fig. 4B, purple shades). In contrast, in the Low-Suppression group (Fig. 4C), the same comparison revealed no NT vs. T decoding accuracy differences. In the High-Suppression group, the observed differences may reflect an early disruption of cue-to-memory conversion processes occurring at around 500 ms. Besides the within-group differences, there were no significant between-group differences in either TNT conditions (Fig. S7c). Note that when using a data-driven K-means clustering method to categorize participants into high- and low-suppression subgroups, we obtained results that are highly similar to the results reported in Fig. 4 (see Fig. S8). Together, these results highlight the important role of an early, active suppression process in successful suppression-induced forgetting.

### Theta and alpha oscillations track item-level perception and reinstatement processes, respectively

We sought converging evidence for the active suppression of individual memories by tracking item-specific oscillatory activity in the theta and alpha bands. Theta and alpha activities have been implicated in perceptual and memory-related processes, such that theta may reflect sensory intake and hippocampo-cortical communication loops (Colgin 2013; Bastos et al. 2015), and alpha may index associative memory and episodic retrieval processes (Hanslmayr et al. 2012a, 2012b; Martín-Buro et al. 2020). If so, posterior theta activity may enable item-specific decoding of the cue objects themselves, whereas alpha activity may enable decoding of reinstated scenes.

In all 3 conditions, we found that theta activity in the 0 to 500 ms window over occipital regions significantly distinguished among individual items, consistent with theta’s putative role in visual processing of individual cue objects (*P*s_corrected_ < 0.001, Fig. S9a to c, also see Fig. S9d to g). During the 500 to 3000 ms window in which scene recollection could unfold, both theta and alpha power drove significant decoding accuracy during Think trials (theta: *P*s_corrected_ < 0.027; alpha: *P*s_corrected_ < 0.039, Fig. S9d). Critically, however, retrieval suppression during No-Think trials abolished any evidence for item-specific decoding based on theta or alpha band activity (Fig. S9e). There was short-lived theta-driven decoding in Perceptual Baseline trials, which may reflect occasional perceptual processing of objects (theta: *P*s_corrected_ < 0.011, Fig. S9f). Channel searchlight analyses during the 500 to 3000 ms window revealed that *alpha* activity over the occipital-parietal region contributed to decoding performance in the Think condition, but did not in either the No-Think or Perceptual Baseline conditions (see Fig. S9h). These findings support the possibility that alpha activity is linked with scene-specific memory reinstatement processes and not simply to object perception. If so, the lack of significant alpha-based decoding in No-Think trials reflects the abolition of memory reinstatement processes arising because of active suppression.

### RSA supports staged cued-recall in Think and the suppression of retrieval in No-Think

**Fig. 5:**
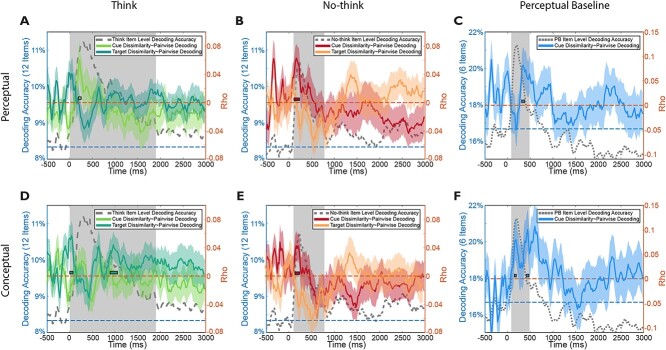
RSA. A to C) Perceptual dissimilarity RSA revealed significant positive correlations during the 0 to 500 ms window between cue perceptual dissimilarity rating and item-level pairwise decoding in all 3 conditions. The correlation coefficients between item-level pairwise decoding accuracy and subjective rating of perceptual dissimilarity rating between items (object cues/target scenes in Think and No-Think, objects in Perceptual Baseline) are plotted on the right *y* axis with colors (Think: green; No-Think: red; Perceptual Baseline: blue), with same-colored disks indicating significant time periods (uncorrected). D to F) Conceptual dissimilarity RSA revealed significant positive correlations on 500 to 1500 ms window between target conceptual dissimilarity rating and item-level pairwise decoding, only in the think condition. The correlation coefficients between item-level pairwise decoding accuracy and subjective rating of conceptual dissimilarity rating between items (object cues/target scenes in Think and No-Think, objects in Perceptual Baseline) are plotted on the right *y* axis with colors (Think: green; No-Think: red; Perceptual Baseline: blue), with same-colored disks indicating significant time periods (uncorrected). Significant item-level decoding accuracies of each condition were plotted on the left *y* axis in gray dashed lines, with gray shade areas indicating significant time periods (same as in Fig. 3A to C).

To further investigate the representational nature underlying item-level decoding results, we collected subjective perceptual and conceptual dissimilarity ratings from an independent group of participants. We used another sample of participants’ subjective ratings to construct a subjective RDM, which was then correlated with the neural RDM consisting of pairwise item-level EEG decoding results. We found significant positive correlations between *cue perceptual* RDM and the neural RDM before 500 ms in all 3 conditions (Think, No-Think, Perceptual Baseline, Fig. 5A to C). This corroborates the notion that significant item-level decoding prior to 500 ms reflect *perceptual* processing of cues, regardless of retrieval conditions.

Notably, we found significant positive correlations between *target conceptual* RDM and the neural RDM around 1000 ms only in the Think condition (Fig. 5D, darker green line), suggesting that the item-level decoding during the later time windows in the retrieval condition was driven by conceptual processing of the retrieved scenes. Notably, this time window overlapped with our hypothesized scene reinstatement window identified in the time-resolved item-level decoding analysis. Meanwhile, no correlations were found between either cue perceptual/conceptual RDMs after 500 ms, arguing against the possibility that the decoding results may capture sustained attention towards cue. We noticed that the RSA correlations were unexpectedly high during the pre-stimulus baseline, which could be because of random fluctuations of item-level decoding accuracies during this time window (even though the mean decoding accuracy equals the chance level).

Together, RSA results supported the staged cued recall in Think trials such that the <500 ms decoding reflected perceptual-related cue processing, whereas the 500 to 1000 ms decoding represented conceptual-related processing of the scene memories. In contrast, retrieval suppression selectively weakened the retrieval of scene memories in No-Think, diminishing both item-level decoding accuracies and their correlations with the target conceptual RDM during the reinstatement window.

## Discussion

Suppressing memory retrieval requires effort; it is not simply neglecting to engage retrieval when an unwelcome reminder appears, but instead involves an active inhibition process (Wimber et al. 2015; Anderson and Hulbert 2021). However, how active suppression unfolds over time to impact individual memories remains poorly understood. Applying multivariate pattern analyses during the eTNT task, we observed, for the first time, the dynamics of how individual aversive memories are suppressed. We found that effective forgetting is associated with (i) the rapid deployment of inhibitory control in suppressing cortical patterns within the first 500 ms, supported by enhanced midfrontal theta activity during efforts to stop retrieval; and (ii) abolished item-specific cortical EEG patterns reflected in the spatial pattern of theta and alpha activity during the 500 to 3000 ms window. This precise chronometry provides new knowledge about the time windows and neural activity critical to achieving successful forgetting via retrieval suppression.

Three findings suggest that an early, active control process truncates retrieval of individual memories, facilitating later forgetting. First, when a reminder cue appeared, within 500 ms retrieval suppression enhanced frontocentral N450 and mid-/right-prefrontal theta activity relative to active retrieval and also relative to a perceptual baseline condition in which scene retrieval was impossible. Given evidence linking frontocentral N450 and frontal midline theta and inhibitory control, particularly during retrieval suppression (Bergström et al. 2009; Cavanagh and Frank 2014; Crespo-García et al. 2022; Mecklinger et al. 2009; Nigbur et al. 2011; Waldhauser et al. 2015), this finding is consistent with the possibility that attempts to stop the retrieval process engaged early inhibitory control. This finding suggests a rapid onset of inhibitory control in the face of an unwelcome reminder, but does not, by itself, link that control process to the successful exclusion of unwanted memories from awareness.

Second, whereas we detected significant item-specific brain activity during active retrieval, retrieval suppression reduced the ability to detect individual items during the 360 to 600 ms time window. The ability to detect reduced item-specific activity in such an early time window indicates that suppression rapidly targets individual unwanted memories and interrupts their retrieval. Estimates based on intracranial EEG recordings suggest that beginning at around 500 ms, hippocampus-dependent pattern completion would normally trigger cortical reinstatement of target memories, accompanied by vivid recollection (Lavenex and Amaral 2000; Colgin 2016; Staresina and Wimber 2019). Given this timing, successful retrieval suppression ideally should be engaged prior to this time window to preempt or truncate the cue-to-memory conversion processes, preventing memories from being reinstated. However, these findings themselves do not link early reductions in cortical reinstatement to later forgetting of the suppressed content.

Third, reduced item-level decoding accuracies during this early time window was associated with later suppression-induced forgetting. Specifically, whereas High-Suppression participants exhibited significantly reduced item-level decoding accuracies during suppression, compared with retrieval in the 280 to 620 ms window, Low-Suppression participants did not. This links the early engagement of control to an increased capacity to forget the suppressed content. Given that hippocampus-dependent pattern completion processes emerge at around 500 ms (Staresina and Wimber 2019), this finding again suggests that successful forgetting depends on rapidly deployed top-down inhibitory control before or during the cue-to-memory conversion time window, preventing cortical reinstatement that would occur in later time windows.

Whereas rapid control significantly attenuated item-level decoding accuracies at around 500 ms, sustained control also occurs during retrieval suppression. Specifically, during the 500 to 3000 ms time window after suppression cue onset, on average MVPA could no longer identify item-specific EEG patterns, yielding findings similar to those in the no-retrieval perceptual baseline condition. The maintenance of control over this longer time period was associated with reduced alpha power throughout the trial (e.g. Waldhauser et al. 2015; Satish et al. 2022). Together, these temporal characteristics reveal a timeline for the suppression of aversive scenes: early control processes truncate retrieval during the perception-to-memory conversion time window (e.g. ~ 360 to 600 ms), with sustained control processes downregulating and eventually abolishing item-specific cortical patterns during the memory reinstatement window until the end of the trial (~800 to 3000 ms).

Because both the object and the scene for each pair are unique, relative to all other pairs, we considered the possibility that our item-level decoding results during voluntary retrieval (i.e. Think) might not reflect reinstatement of the associated memory, but rather reflect sustained covert attention to individual object. Three features of our findings argue against this possibility, however. First, we found that the early (0 to 500 ms) vs. late (500 to 3000 ms) decoding patterns during Think trials were characterized by distinct EEG spatial distributions, suggesting a qualitative shift in processing over time, consistent with perception-to-memory staged recall model (Staresina and Wimber 2019). Second, we found that only decoding accuracy during the 500 to 3000 ms, but not the 0 to 500 ms window, tended to predict better recall of Think items on the later memory test. Third, our RSA provided additional evidence that only the early (0 to 500 ms) time window reflected perceptual processing of the cue object. Despite the extended presentation of the cue object, the item-specific EEG decoding patterns derived from later time windows during retrieval were more strongly associated with participants’ conceptual similarity judgments than with their perceptual judgments on cue objects. Thus, converging evidence from different analytical approaches suggests that whereas the early EEG decoding patterns reflect perceptual processes acting on item-specific cues, the later EEG decoding patterns during Think likely reflect recollection of the accompanying scene. These results and the timelines found here are highly consistent with research in the neural chronometry of cued recall (Staresina and Wimber 2019).

Consistent with this interpretation, both theta and alpha power contributed to item-level decoding during voluntary retrieval, with an early onset of occipital theta activity followed by parietal-occipital alpha activity. Theta and alpha activities have been implicated in perceptual and memory-related processes, such that theta may reflect sensory intake and hippocampo-cortical communication loops (Colgin 2013; Bastos et al. 2015). Relatedly, linking behavioral oscillation and neural oscillation, a recent study demonstrated a prominent role of theta rhythm in memory retrieval (Ter Wal et al. 2021). Decoding patterns during Perceptual Baseline trials provided converging support for this account: when participants viewed object cues that lacked any associated scene memory, only occipital theta activity in the 0 to 500 ms window drove significant item-level decoding, ruling out any contribution of scene retrieval.

If the foregoing staged view of retrieval is correct, then item-specific decoding based on alpha-band activity after initial cue processing may reflect the reinstatement of individual scenes (Staresina et al. 2019; Staresina and Wimber 2019). For example, in a directed forgetting task, Fellner et al. (2020) reported alpha power increases 1000 to 2000 ms following to-be-remembered cues, which were associated with selective rehearsal processes. Mirroring this result, we found that compared with No-Think or Perceptual Baseline trials, Think condition enhanced alpha power during the same 1000 to 2000 ms window when reinstatement of the associated scene would be expected (Fig. S5). This result may appear inconsistent with the influential Sync/desync model, which posits that alpha power reduction was associated with successful memory formation and retrieval (e.g. Hanslmayr et al. 2012a, 2016). Moreover, previous TNT research also showed that Think trials were associated with alpha power reduction within 1000 ms post-cue (Depue et al. 2013). However, it should be noted that our Think trials required participants to maintain the complex negative scenes in mnemonic awareness while shielding distracting information from the external environment, which could increase alpha power (see Bäuml et al. 2008; Bonnefond and Jensen 2012; Xie et al. 2020). Relatedly, our results are highly consistent with many prior TNT studies, such that No-Think trials were associated with sustained alpha power reduction relative to Think trials (see Waldhauser et al. 2015; Legrand et al. 2020; Satish et al. 2022). In particular, the relative reduction of alpha power during No-Think trials may reflect active downregulation and sustained control of unwanted memories, together with weakened/abolished memory reinstatement throughout the 500 to 3000 ms time window. These results highlight the importance of interpreting alpha power dynamics in memory tasks considering the experimental design/instructions and the specific cognitive processes.

Whereas our results suggest that the 500 to 3000 ms Think vs. No-Think differences in item-specific decoding patterns reflected suppression of individual memories, it is possible that different levels of attention to cues during this period also contributed to observed effects. Although we strongly emphasized that participants should pay full and sustained attention to cues regardless of retrieval conditions, participants might have disengaged from object cues in No-Think and Perceptual Baseline, once they figured out that retrieval was not necessary. Thus, variations in attention devoted to object cues could contribute to the Think vs. No-Think differences in item-specific decoding accuracies. However, the Think vs. No-Think item-level decoding differences were most evident among High-Suppression participants, suggesting the decoding accuracies were linked with the behavioral aftereffects of retrieval vs. retrieval suppression. Furthermore, correlating the cue-/target-related perceptual/conceptual RDMs with the neural RDMs during 500 to 3000 ms, the RSA results showed that while significant decoding in Think trials were associated with scene-related conceptual processing, there was no evidence of scene-related processing during No-Think trials. This additional evidence supports the argument that the item-level decoding patterns in the later time window may reflect target scene-related retrieval processing, rather than the cue object-related attentional variations.

Despite of these supporting evidence, one limitation of our experiment is that we did not record EEGs during the encoding phase, nor include a separate object-vs.-scene functional localizer task. Future research shall include such functional localizer tasks to better capture cue- and target-specific neural representations and the cue-to-memory conversion processes (e.g. Treder et al. 2021; Legrand et al. 2022). Moreover, despite the advantage of EEG multivariate analyses, the relatively poor spatial resolution of scalp EEG did not allow us to delineate the specific brain regions involved. To overcome this limitation, techniques such as simultaneous EEG and fMRI recording (e.g. Crespo-Garcia et al. 2022) or intracranial EEG (Ten Oever et al. 2021) that afford both high temporal and spatial resolution bear promises in revealing the precise spatial-temporal neural dynamics underlying retrieval suppression.

Another open question is the extent to which the observed temporal patterns in retrieval and retrieval suppression could be generalized to non-aversive memories. Given that we used aversive scenes to better simulate the control of real-life unwanted memories, the observed decoding results and the temporal dynamics could be specific to negative scene memories. However, our hypothesis on the temporal dynamics of retrieval suppression is largely guided by the cued recall chronometry research (Yaffe et al. 2014; Staresina et al. 2019; Staresina and Wimber 2019; Treder et al. 2021), which mostly uses neutral materials such as words or object images. It is thus plausible that the results observed here could be generalized to other materials, such as neutral pictures. Future research can directly compare the temporal dynamics of suppressing individual aversive vs. non-aversive memory to test this possibility.

Taken together, our findings show that for successful retrieval suppression and forgetting, inhibitory control needs to be deployed rapidly. On the one hand, early enhanced frontal theta disrupted cue-to-memory conversion, truncating the reinstatement of individual aversive scene memories within the first 500 ms upon seeing the cues. On the other hand, sustained control weakened and eventually abolished item-specific cortical EEG patterns during the 500 to 3000 ms time window, reflected in reduced alpha activity. In contrast, diminished early control compromised successful voluntary forgetting of suppressed content. By tracking the precise timing and neural dynamics of retrieval suppression in modulating individual memories, our results may inform future research on when and how to intervene during retrieval suppression to improve people’s ability to forget unwanted memories.

## Author contributions

Xuanyi Lin (Conceptualization, Data curation, Formal analysis, Investigation, Methodology, Visualization, Writing—original draft, Writing—review & editing), Danni Chen (Formal analysis, Writing—review & editing), Jing Liu (Formal analysis, Writing—review & editing), Ziqing Yao (Formal analysis, Writing—review & editing), Hui Xie (Formal analysis, Writing—review & editing), Michael C. Anderson (Conceptualization, Formal analysis, Writing—original draft, Writing—review & editing), and Xiaoqing Hu (Conceptualization, Formal analysis, Funding acquisition, Project administration, Resources, Supervision, Writing—original draft, Writing—review & editing).

## Funding

X.H. is supported by the Ministry of Science and Technology of China STI2030-Major Projects (No. 2022ZD0214100), National Natural Science Foundation of China (No. 32171056), General Research Fund (No. 17614922) of Hong Kong Research Grants Council. M.C.A. is supported by UK Medical Research Council grant MC-A060-5PR00.

*Conflict of interest statement*: None declared.

## Data and code availability

Preprocessed behavioral/EEG data and analysis scripts are deposited at OSF: https://osf.io/6tr4g/?view_only=e8b96afa183044a781aa8b8610735743.

## Supplementary Material

Lin_TNT_CC_R1_MS_SOM_Submit_bhae080
