# Supplementary material for: Observing the suppression of individual aversive memories from conscious awareness
Source: Cereb Cortex. 2024 Jun 11;34(6):bhae080. doi: 10.1093/cercor/bhae080 (PMC11166503; doi:10.1093/cercor/bhae080)
Supplement: Lin_TNT_CC_R1_MS_SOM_Submit_bhae080 [file lin_tnt_cc_r1_ms_som_submit_bhae080.docx]

**Supplemental Materials**


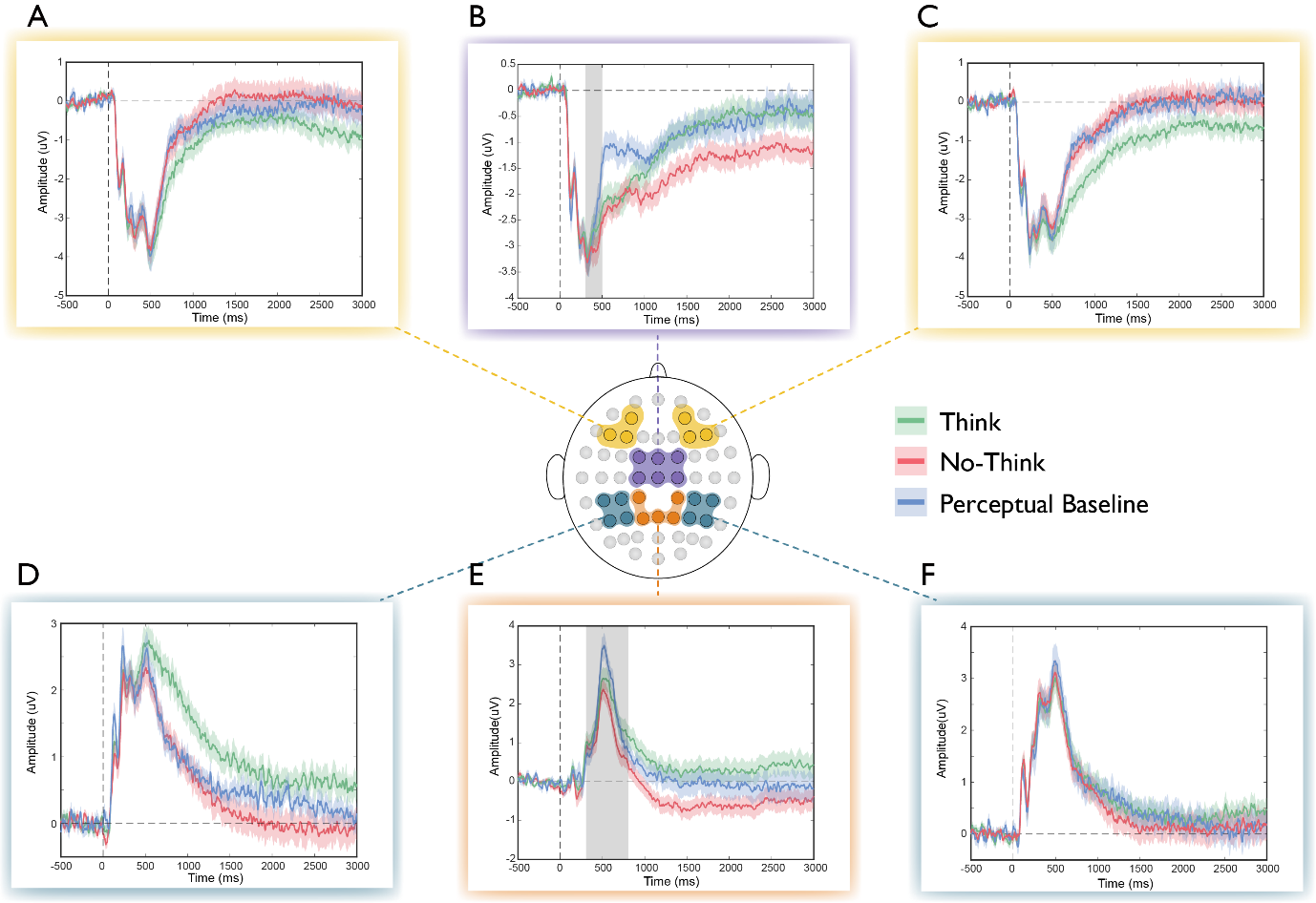


Figure S1. ERP Results

(A, C) ERPs averaged at channels over left and right prefrontal regions: left-prefrontal region includes AF3, F3, F5; right- prefrontal region includes AF4, F4, F6. The ERP index of working memory maintenance, NSW, was lower in No-Think compared to Think. A 3 (condition: Think vs. No-Think vs. PB) by 3 (region: left- vs. central vs. right-parietal regions) within-subjects ANOVA revealed a significant two-way interaction of condition and electrode region, *F*(1.69,65.86) = 5.90, p = .007, ηp2 = .131. Follow-up paired-sample t-tests showed that No-Think trials elicited significantly reduced NSW than Think trials in both left prefrontal clusters, t(39) = -3.53, p = .001, dz = 0.56; and right prefrontal clusters, t(39) = -4.98, p < .001, dz = 0.79. When compared to PB trials, No-Think trials significantly reduced NSW over the left prefrontal clusters, t(39) = -2.69, p = .010, dz =0.43; but not over the right prefrontal area, t(39) = -0.57, p = .570, dz = 0.09.

(B) ERPs averaged at channels over frontal-central region (Fz, F1, F2, Cz, C1, C2). The ERP index of cognitive control, N450, was enhanced in No-Think compared to Think.

(D-F) ERPs averaged at channels over three parietal regions: left-parietal (CP3, CP5, P3, P5), central-parietal (CP1, CP2, Pz, P1, P2), and right-parietal (CP4, CP6, P4, P6). The ERP index of memory retrieval, P300, was reduced in No-Think compared to Think.

**
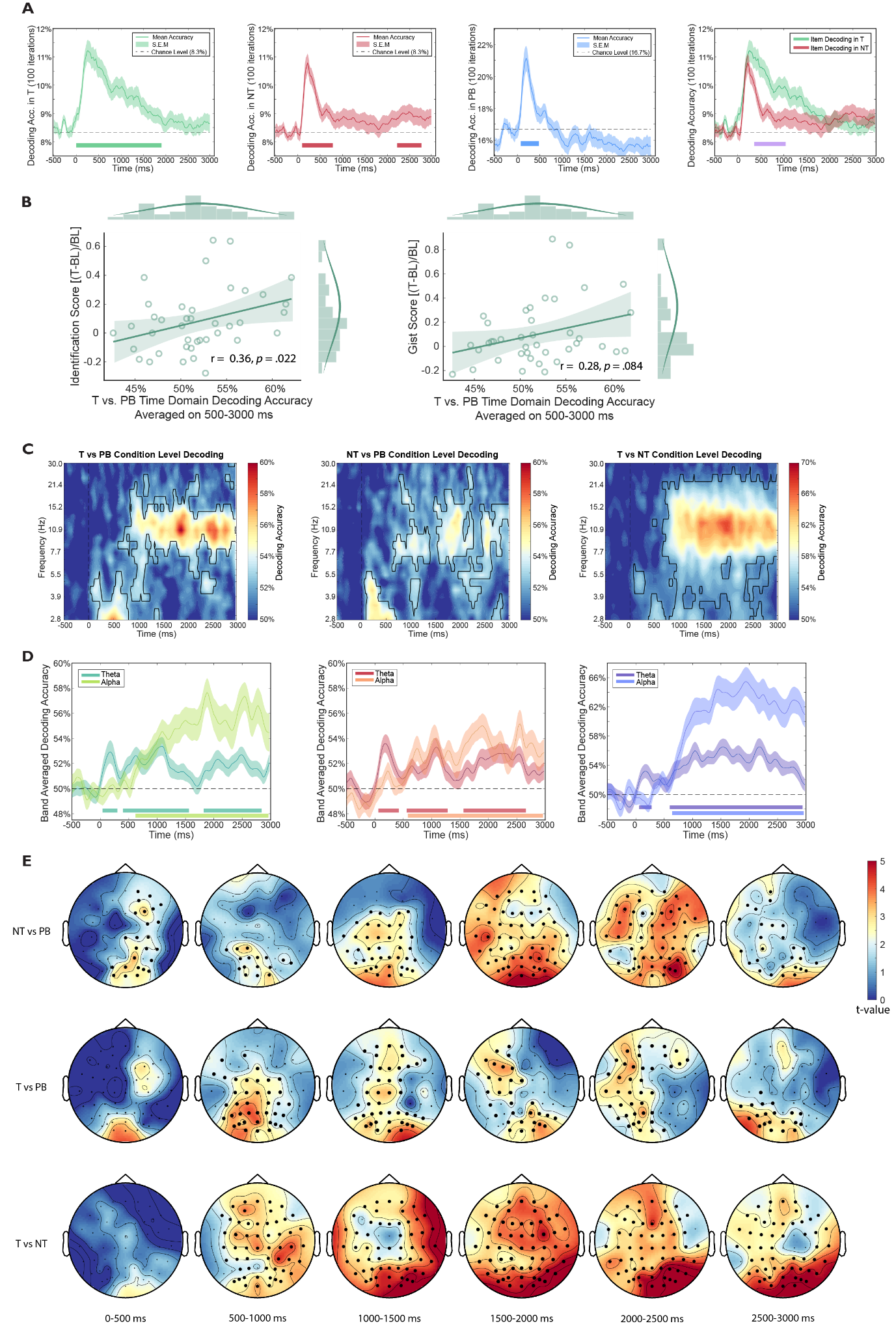
**

Figure S2. Item-Level Time Domain EEG Decoding with 100 iterations, Individual Difference Results and Condition Level Theta Searchlight.

(A) Item-level time domain-decoding was validated with 100 iterations. The results were highly similar to those reported in main text (using 10 iterations). Moreover, the comparison between *T* and *NT* item level decoding found a significant cluster on 360-1040 ms, which indicated this effect was robust and even larger when run with higher iterations.

(B) Time domain Think vs. Perceptual Baseline decoding accuracies during the 500-3000 ms window was positively correlated with the above-baseline enhancement of memory recall in the Think condition, based on the Identification and Gist score ((Think – Baseline)/Baseline in the final cued recall test, i.e. the retrieval benefit, proportional to baseline.

(C) Condition-level time-frequency decoding results. Frequency is log scaled with the colorbar denoting decoding accuracy. Black outlined areas highlight significant clusters against chance level (both cluster and permutation α are set at 0.05).

(D) Decoding accuracies in A-C are averaged on theta (4-8 Hz) and alpha (9-12 Hz) bands. Lines at the bottom denote significant clusters of averaged accuracy against chance level (50%) with permutation correction. (E) Condition-level searchlight decoding using theta power averaged on every 500 ms on the whole epoch. From top to bottom rows are NT vs. PB, T vs. PB, and T vs. NT decoding. From left to right are the six time-windows from 0 to 3000 ms. Significant electrodes were cluster corrected and are highlighted. Theta power on 0-500 ms distinguished NT vs. PB over frontal and posterior brain regions.


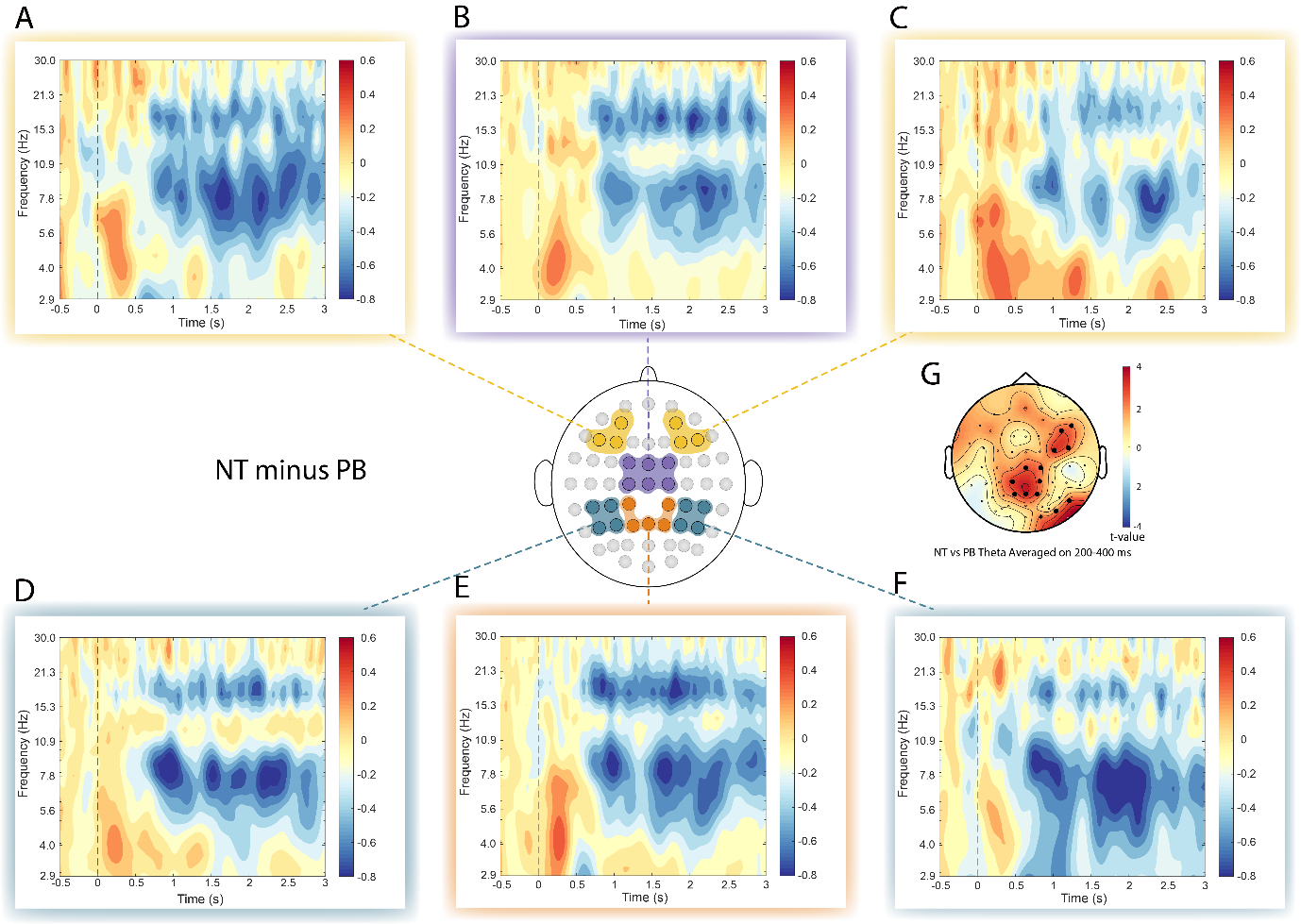


Figure S3. No-think vs. Perceptual Baseline Time Frequency Results.

(A, C) No-Think minus Perceptual Baseline TFRs averaged at channels over left and right Prefrontal regions: left-prefrontal region includes AF3, F3, F5; right- prefrontal region includes AF4, F4, F6.

(B) No-Think minus Perceptual Baseline TFRs averaged at channels over Frontal-central region (Fz, F1, F2, Cz, C1, C2).

(D-F) No-Think minus Perceptual Baseline TFRs averaged at channels over three Parietal regions: left-parietal (CP3, CP5, P3, P5), central-parietal (CP1, CP2, Pz, P1, P2), and right-parietal (CP4, CP6, P4, P6).

(G) Theta oscillations averaged on 200-400 ms increased in No-Think vs. Perceptual Baseline over prefrontal- and central parietal regions. Electrodes with significant difference are highlighted (cluster-corrected).
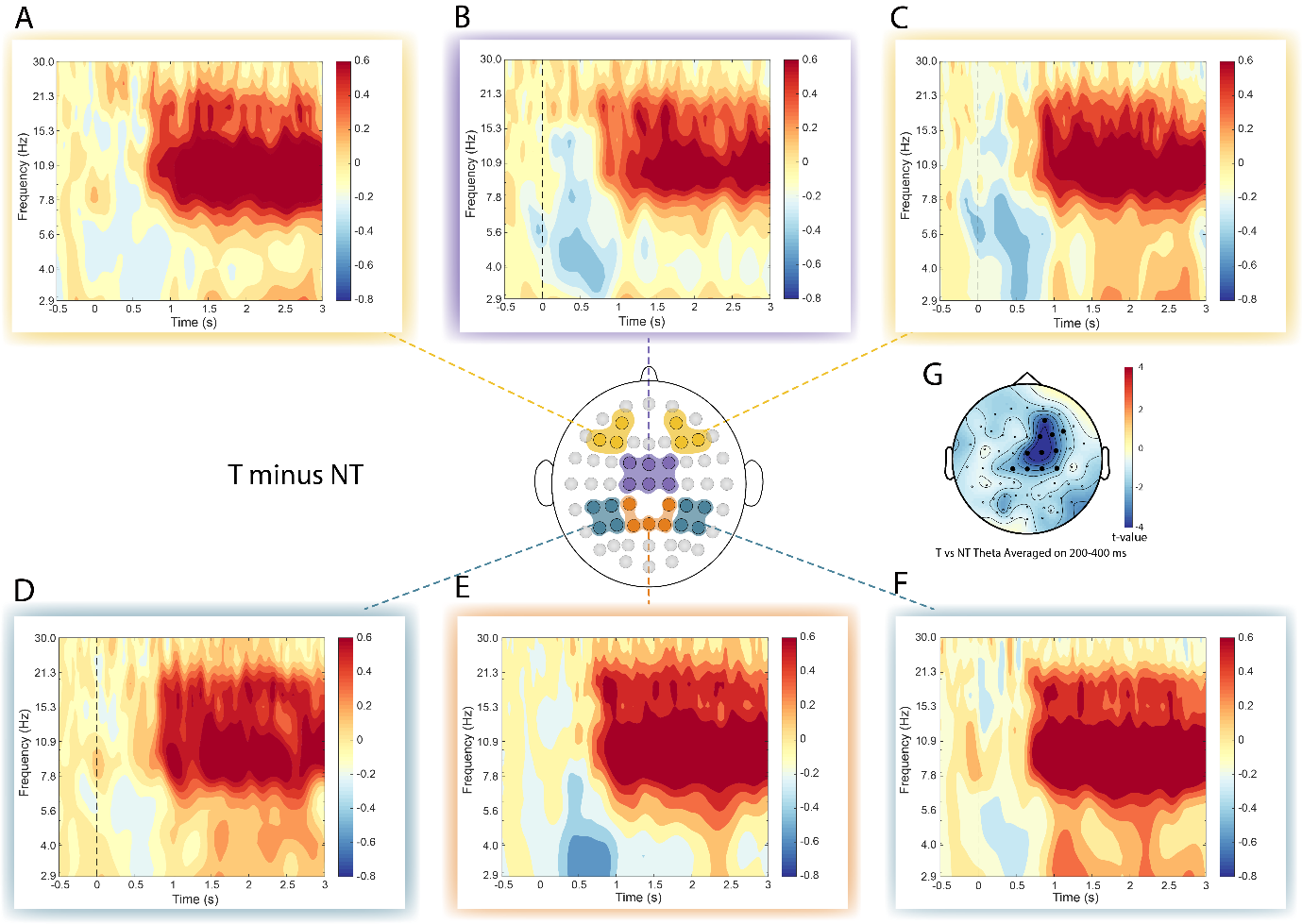


Figure S4. Think vs. No-think Time Frequency Results.

(A, C) Think minus No-think TFRs averaged at channels over left and right Prefrontal regions: left-prefrontal region includes AF3, F3, F5; right- prefrontal region includes AF4, F4, F6.

(B) Think minus No-think TFRs averaged at channels over Frontal-central region (Fz, F1, F2, Cz, C1, C2).

(D-F) Think minus No-think TFRs averaged at channels over three Parietal regions: left-parietal (CP3, CP5, P3, P5), central-parietal (CP1, CP2, Pz, P1, P2), and right-parietal (CP4, CP6, P4, P6).

(G) Theta power averaged from 200-400 ms was higher in NT than T. The decreased theta power showed a frontal-central distribution. Significant electrodes were cluster corrected and are highlighted.


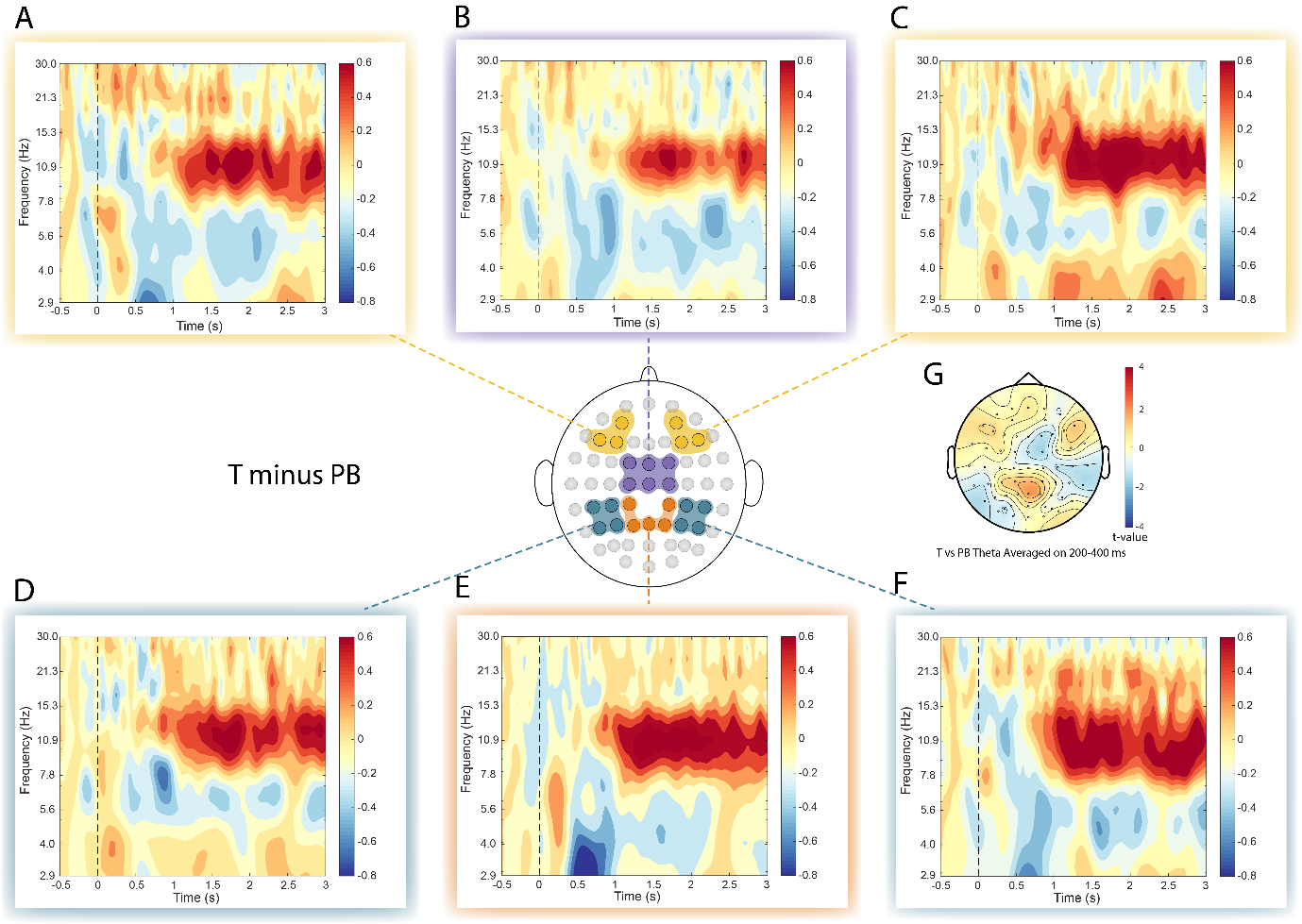


Figure S5. Think vs. Perceptual Baseline Time Frequency Results.

(A, C) Think minus Perceptual Baseline TFRs averaged at channels over left and right Prefrontal regions: left-prefrontal region includes AF3, F3, F5; right- prefrontal region includes AF4, F4, F6.

(B) Think minus Perceptual Baseline TFRs averaged at channels over Frontal-central region (Fz, F1, F2, Cz, C1, C2).

(D-F) Think minus Perceptual Baseline TFRs averaged at channels over three Parietal regions: left-parietal (CP3, CP5, P3, P5), central-parietal (CP1, CP2, Pz, P1, P2), and right-parietal (CP4, CP6, P4, P6).

(G) Theta oscillations averaged on 200-400 ms between Think vs. Perceptual Baseline were comparable. Electrodes with significant difference are highlighted (cluster-corrected).


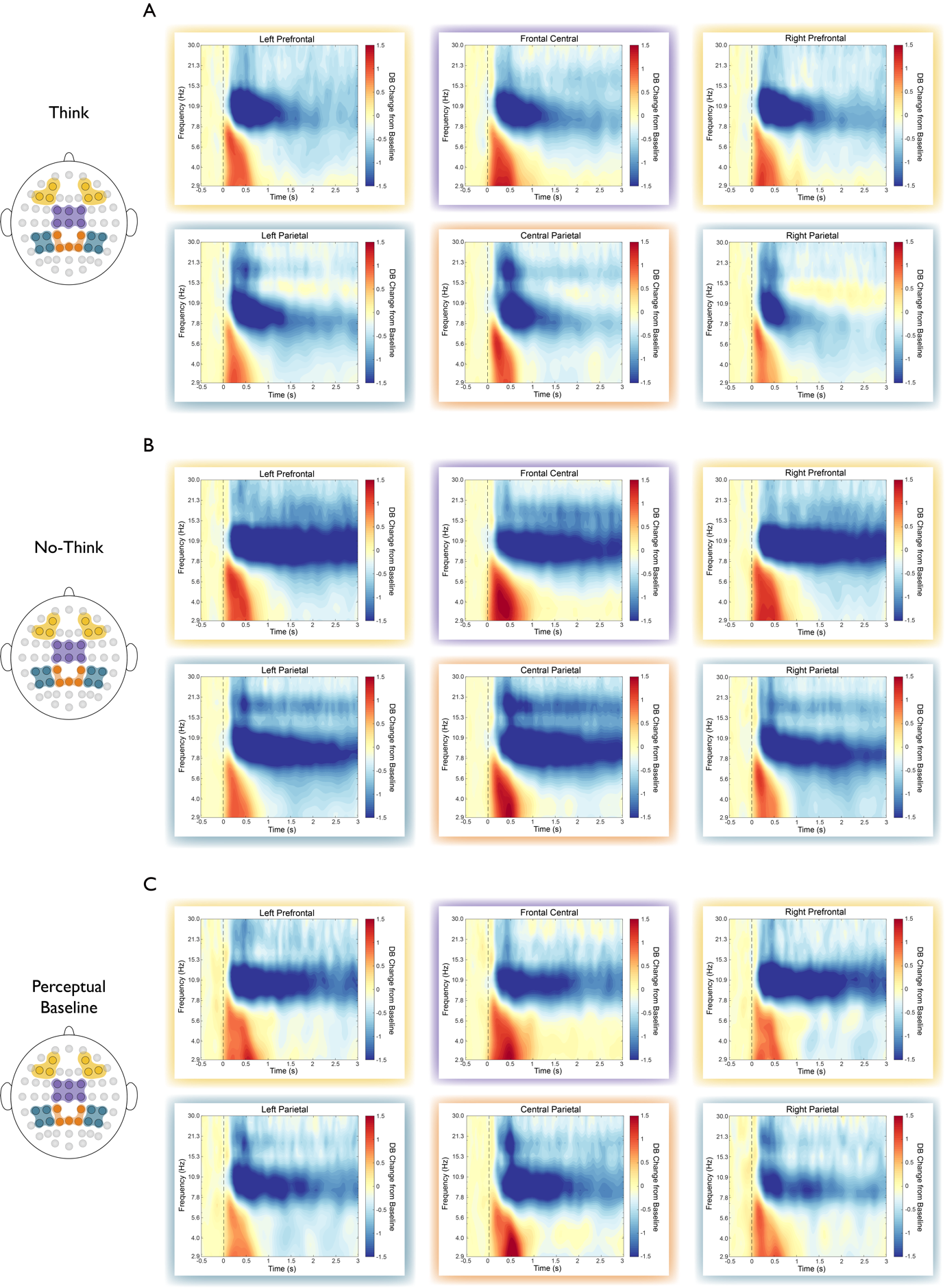


Figure S6. Time-frequency representations of each condition

(A) Think; (B) No-think; (C) Perceptual Baseline.


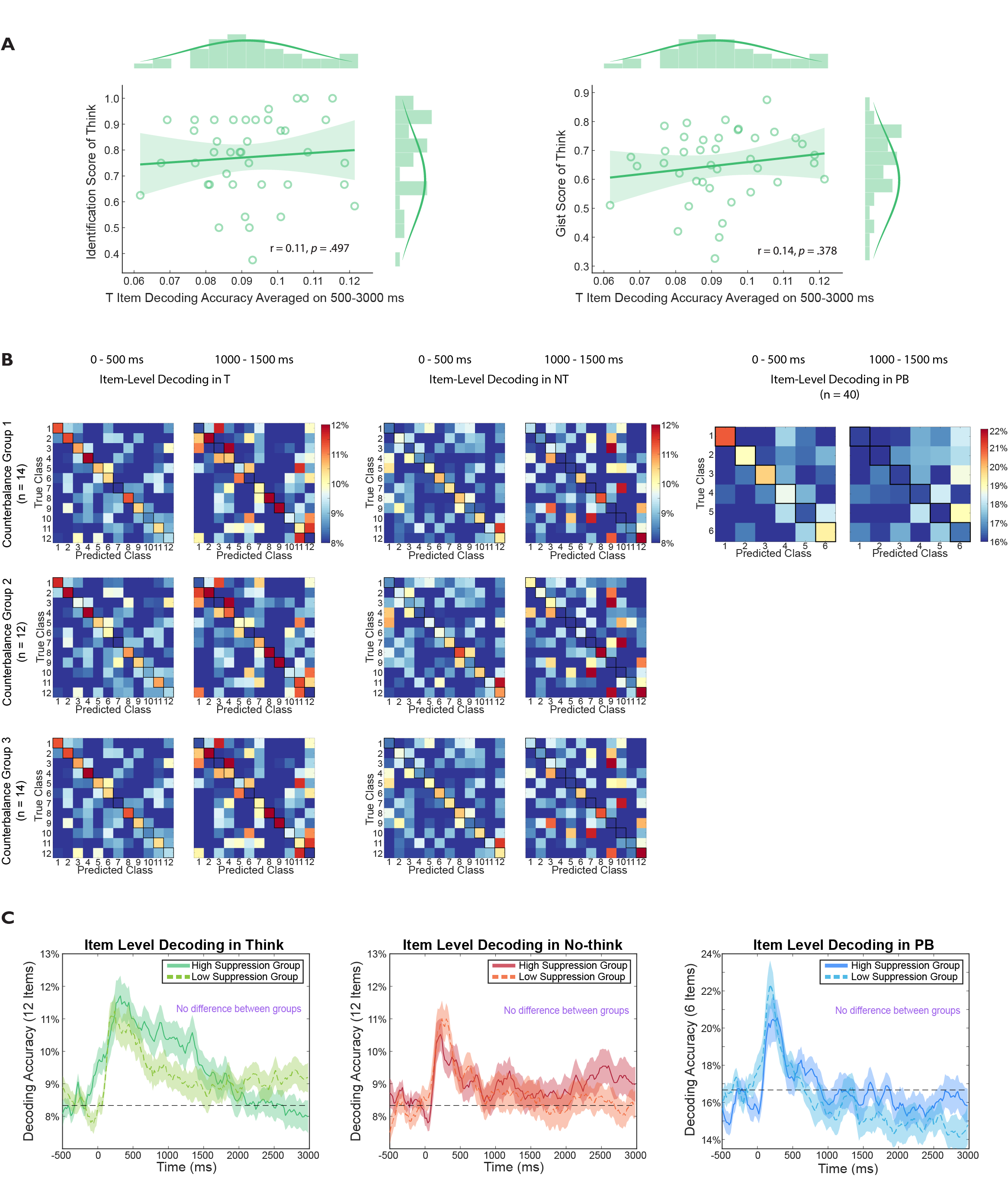


Figure S7. Item-Level Time Domain EEG Decoding Correlation, Confusion Matrices and High- vs. Low-Suppression Group.

(A) Correlations between item-level decoding accuracy in Think and Gist/Identification memory.

(B) Confusion matrices of item-level decoding accuracies averaged on 0-500 ms and 1000-1500 ms. In *T* & *NT*, items were divided into three counterbalance groups and their confusion matrices were plotted separately. No counterbalance was performed for Perceptual Baseline.

(C) Comparisons of item-level decoding results between High- and Low-Suppression in each of the three conditions. No between group differences were found in any of the three conditions.


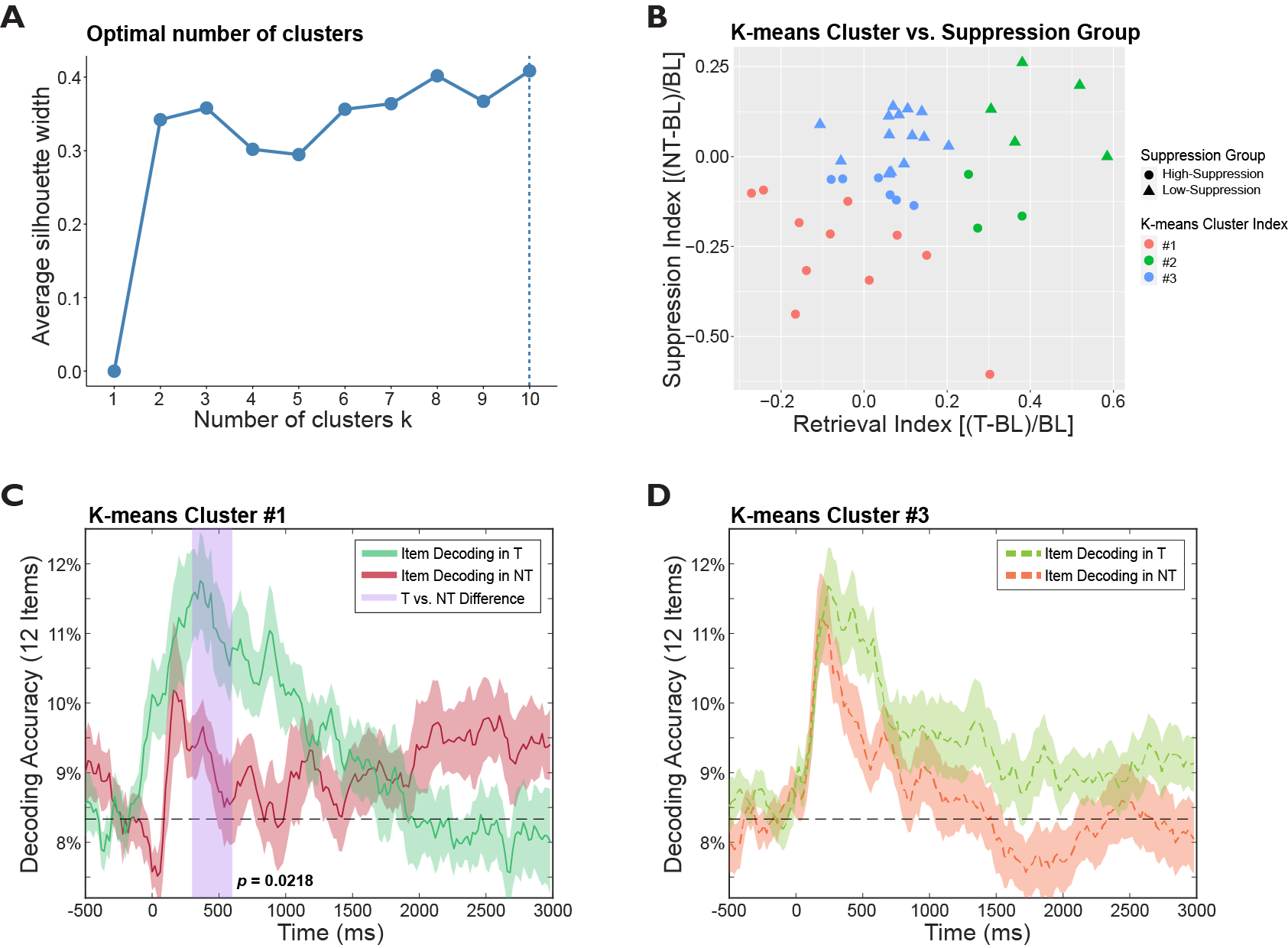


Figure S8. K-means clustering High-/Low-Suppression Sub-groups.

1. Silhouette plot of k-means clustering result.
2. Comparison between K-means clustering (k = 3) and median-split grouping.
3. Comparison between Think and No-Think item-level decoding among participants in #1 K-means cluster. Time windows with significant differences were marked by purple shaded area (*p* = .0218, permutation corrected).
4. Comparison between Think and No-think item-level decoding among participants in #3 K-means cluster. No significant differences were observed. Both cluster and permutation alpha are 0.05, with one-sided comparisons against chance level.


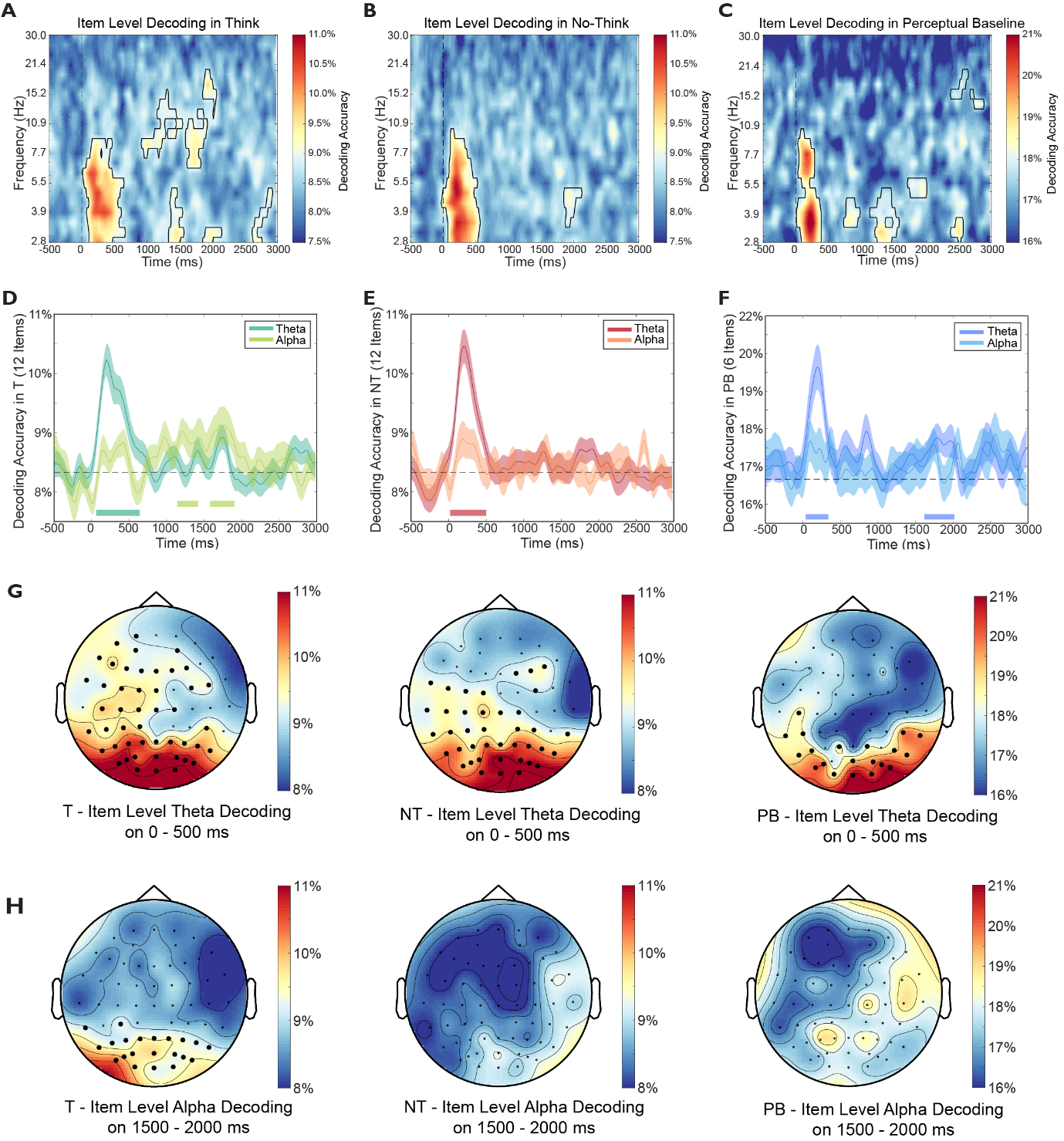


Figure S9. Item-level Time-Frequency Domain Decoding.

(A-C) Item-level time-frequency decoding results. Frequency is log scaled and the colorbar denotes decoding accuracy. The black outline highlights significant clusters against chance levels (both cluster alpha and permutation α are 0.05, one-sided).

(D-F) Decoding accuracies in A-C are averaged on theta and alpha bands. Horizontal bars denote significant clusters of the band-averaged accuracies against chance level (cluster corrected, one-sided αs = 0.05).

(G) Item-level theta searchlight during the 0-500 ms window showed an occipital distribution in all three conditions. Significant channels are highlighted (permutation cluster corrected with one-sided αs = 0.05).

(H) Item-level alpha searchlight during the 1500-2000 ms window showed that only in the Think condition was alpha power able to distinguish among items. The alpha searchlight decoding in the Think condition originated from the posterior region. Significant channels are highlighted (permutation cluster corrected with one-sided αs = 0.05).
